# The Probability of a Gene Tree Topology within a Phylogenetic Network with Applications to Hybridization Detection

**DOI:** 10.1371/journal.pgen.1002660

**Published:** 2012-04-19

**Authors:** Yun Yu, James H. Degnan, Luay Nakhleh

**Affiliations:** 1 Department of Computer Science, Rice University, Houston, Texas, United States of America; 2 Department of Mathematics and Statistics, University of Canterbury, Christchurch, New Zealand; 3 National Institute of Mathematical and Biological Synthesis, Knoxville, Tennessee, United States of America; University of Washington, United States of America

## Abstract

Gene tree topologies have proven a powerful data source for various tasks, including species tree inference and species delimitation. Consequently, methods for computing probabilities of gene trees within species trees have been developed and widely used in probabilistic inference frameworks. All these methods assume an underlying multispecies coalescent model. However, when reticulate evolutionary events such as hybridization occur, these methods are inadequate, as they do not account for such events. Methods that account for both hybridization and deep coalescence in computing the probability of a gene tree topology currently exist for very limited cases. However, no such methods exist for general cases, owing primarily to the fact that it is currently unknown how to compute the probability of a gene tree topology within the branches of a phylogenetic network. Here we present a novel method for computing the probability of gene tree topologies on phylogenetic networks and demonstrate its application to the inference of hybridization in the presence of incomplete lineage sorting. We reanalyze a *Saccharomyces* species data set for which multiple analyses had converged on a species tree candidate. Using our method, though, we show that an evolutionary hypothesis involving hybridization in this group has better support than one of strict divergence. A similar reanalysis on a group of three *Drosophila* species shows that the data is consistent with hybridization. Further, using extensive simulation studies, we demonstrate the power of gene tree topologies at obtaining accurate estimates of branch lengths and hybridization probabilities of a given phylogenetic network. Finally, we discuss identifiability issues with detecting hybridization, particularly in cases that involve extinction or incomplete sampling of taxa.

## Author Summary

Species trees depict how species split and diverge. Within the branches of a species tree, gene trees, which depict the evolutionary histories of different genomic regions in the species, grow. Evolutionary analyses of the genomes of closely related organisms have highlighted the phenomenon that gene trees may disagree with each other as well as with the species tree that contains them due to deep coalescence. Furthermore, for several groups of organisms, hybridization plays an important role in their evolution and diversification. This evolutionary event also results in gene tree incongruence and gives rise to a species phylogeny that is a *network*. Thus, inferring the evolutionary histories of groups of organisms where hybridization is known, or suspected, to play an evolutionary role requires dealing simultaneously with hybridization and other sources of gene tree incongruence. Currently, no methods exist for doing this with general scenarios of hybridization. In this paper, we propose the first method for this task and demonstrate its performance. We revisit the analysis of a set of yeast species and another of *Drosophila* species, and show that evolutionary histories involving hybridization have higher support than the strictly diverging evolutionary histories estimated when not incorporating hybridization in the analysis.

## Introduction

A molecular systematics paradigm that views molecular sequences as the characters of gene trees, and gene trees as characters of the species tree [1] is being increasingly adopted in the post-genomic era [2], [3]. Several models of evolution for the former type of characters have been devised [4], while the coalescent has been the main model of the latter type of characters [5], [6]. However, hybridization, a process that is believed to play an important role in the speciation and evolutionary innovations of several groups of plant and animal species [7], [8], results in reticulate (species) evolutionary histories that are best modeled using a *phylogenetic network* [9], [10]. Further, as hybridization may occur between closely related species, incongruence among gene trees may also be partly due to deep coalescence, and distinguishing between the two factors is hard under these conditions [11]. Therefore, to enable a more general application of the new paradigm, a phylogenetic network model that allows simultaneously for deep coalescence events as well as hybridization is needed [12]. This model can be devised by extending the coalescent model to allow for computing gene tree probabilities in the presence of hybridization. In this paper we focus on gene tree topologies and analyze the signal they contain for detecting hybridization in the presence of deep coalescence.

Applications of probabilities of gene tree topologies given species trees include determining statistical consistency (or inconsistency) of topology-based methods for inferring species trees 13–15, testing the multispecies coalescent model [13], [16], determining identifiability of species trees using linear invariants of functions of gene tree topology probabilities [17], [18], delimiting species [19], designing simulation studies for species tree inference methods [20]–[22], and inferring species trees [23], [24]. We expect that similar applications may be useful for probabilities of gene tree topologies given species networks. In particular, it will be useful to be able to evaluate the performance of methods that infer species trees in the presence of hybridization as well as the performance of methods for inferring species networks. Knowing the distribution of gene tree topologies could also be useful for estimating the probability that two gene trees have the same topology, a quantity that is used in constructing the prior which models gene tree discordance in BUCKy [25], a program that is often used to estimate species trees or concordance trees.

A method for computing the probability mass function of gene tree topologies in the absence of hybridization (i.e., under the multispecies coalescent model is assumed) is given by Degnan and Salter [26]. However, to handle hybridization and deep coalescence simultaneously, this method has to be extended to allow for reticulate species evolutionary histories.

Indeed, attempts have been made recently for this very task [27]–[30], all of which have focused on very limited special cases where the phylogenetic network topology is known and contains one or two hybridization events, and a single allele sampled per species. However, a general formula for the probability of a gene tree topology given a general (any number of taxa, hybridizations, gene trees, and/or alleles) phylogenetic network has remained elusive.

A binary phylogenetic network topology 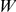 contains two types of nodes: *tree nodes*, each of which has exactly one parent (except for the root, which has zero parents), and *reticulation nodes*, each of which has exactly two parents. The edge incident into a tree node is called *tree edge*, and the edges incident into a reticulation node are called *reticulation edges*. In our context, we associate with a phylogenetic network 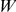 a vector of branch lengths 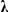 (in units of 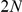 generations, where 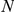 is the effective population size in that branch) and a vector of hybridization probabilities 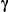 (which indicates for each allele in a hybrid population its probability of inheritance from each of the two parent populations); see Text S1 for formal definition. The gene tree topology 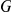 can be viewed as a random variable with probability mass function 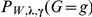. In this paper, we solve the aforementioned open problem by reporting on a novel method for computing the probability of a gene tree topology given a phylogenetic network, 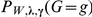.

We illustrate the use of gene tree topology probabilities to estimate the values of species network parameters using the likelihood of the gene tree topologies. This application allows for disentangling hybridization and deep coalescence when analyzing a set of incongruent gene trees, as both events can give rise to similar incongruence patterns. Given a collection 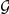 of gene tree topologies, one per locus, in a set of sampled loci, the likelihood function is given by
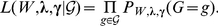
(1)This formulation provides a framework for estimating the parameters 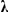 and 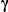 of an evolutionary history hypothesis 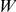, given a collection of gene trees 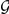. Estimates of 0 or 1 for the entries in the 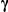 vector reflect the absence of evidence for hybridization based on the gene tree topology distribution.

As gene tree topologies are estimated from sequence data, there is often uncertainty about them. In our method, we account for that in two ways: (1) by considering a set of gene tree topology candidates, along with their associated probabilities (produced, for example, by a Bayesian analysis), and (2) by considering for each locus the strict consensus of all optimal tree topologies computed for that locus (produced, for example, by a maximum parsimony analysis).

Finally, to account for model complexity, we employ a simple technique based on three information criteria, AIC [31], AICc [32] and BIC [33]. While these criteria have their shortcomings for model selection, the question of how to account for phylogenetic network complexity is still wide open and no methods exist for addressing it systematically [10].

We have implemented our method in the publicly available software package PhyloNet [34] and demonstrated its broad utilities in three domains. First, we reanalyze a *Saccharomyces* data set and a *Drosophila* data set, and find support for hybridization in both data sets. Second, we show the identifiability of the parameter values of certain reticulate evolutionary histories. Third, we highlight and discuss the lack of identifiability of the parameters in other scenarios that involve extinctions.

## Materials and Methods

We begin by reviewing Degnan and Salter's method for computing the probability gene tree topologies on species trees, and then describe our novel extension to the case of species networks.

### The probability of a gene tree topology within a species tree

Degnan and Salter [26] gave the mass probability function of a gene tree topology 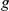 for a given species tree with topology 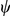 and vector of branch lengths 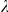 as
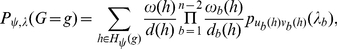
(2)which is taken over coalescent histories 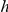 from the set of all coalescent histories 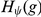. The product is taken over all internal branches 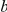 of the species tree. The term 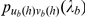 is the probability that 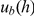 lineages coalesce into 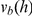 lineages on branch 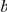 whose length is 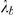. And the terms 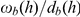 and 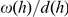 represents the probability that the coalescent events agree with the gene tree topology. In particular, 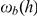 is the number of ways that coalescent events can occur consistently with the gene tree and 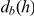 is the number of sequences of coalescences that give the number of coalescent events specified by 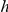. However, this equation assumes that 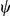 is a tree and as such is inapplicable to reticulate evolutionary histories. Recently, this equation was adapted to very special cases of species phylogenies with hybridization [28]–[30]. However, none of these adaptations is general enough to allow for multiple hybridizations, multiple alleles per species, or arbitrary divergence patterns following hybridization. We present a novel approach for generalizing this equation to handle hybridization. Our approach is general enough in that it allows for computing gene tree probabilities on any binary phylogenetic network topology, thus overcoming limitations of recent works.

### The probability of a gene tree topology within a species network

**Figure 1:**
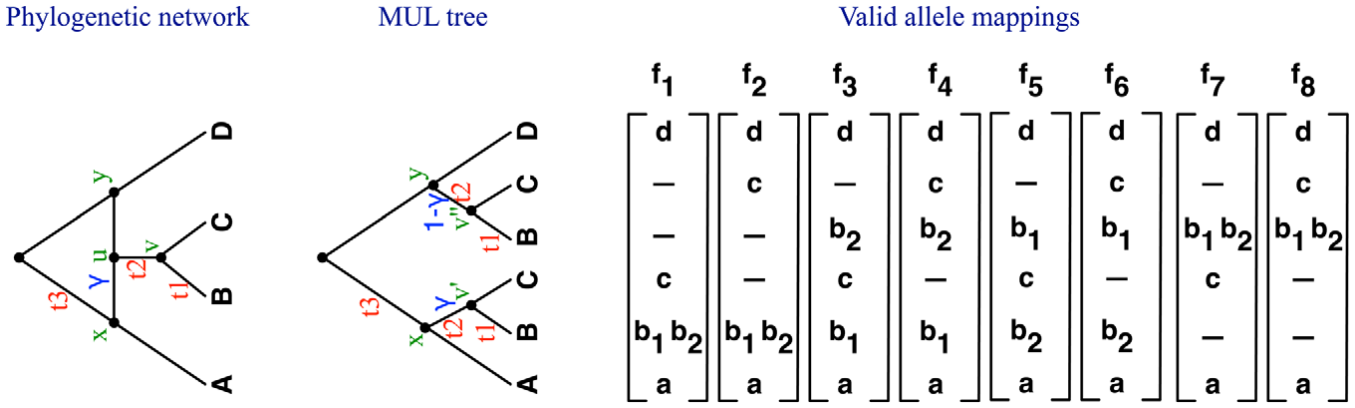
Phylogenetic networks, MUL trees, and valid allele mappings. In this example, single alleles 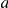, 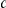, and 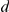 were sampled from each of the three species 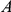, 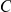, and 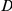, respectively, whereas two alleles (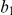 and 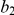) were sampled from species 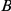. See text and Text S1 for details.

Our approach for computing the probability of a gene tree 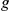 given a species network 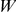 has three steps. First, 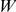 is converted into a multilabeled (MUL) tree 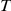 (a tree whose leaves are not uniquely labeled by a set of taxa; see Text S1); second, the alleles at the tips of 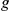 are mapped in every valid way to the tips of 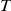; and, finally, the probability of 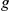 is computed as the sum, over all valid allele mappings, of probabilities of 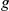 given 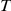 (see Figure 1).

#### Step 1: Converting the phylogenetic network 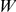 to MUL tree 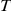


Let 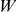 be a phylogenetic network on set 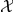 of species, and with branch lengths vector 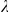 and hybridization probabilities vector 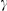. The conversion of 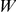 into a MUL tree is done as follows. Traversing the network 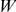 from the leaves towards the root, every time a reticulation node 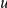 is encountered, the two reticulation edges incident into it are removed, an additional copy of the subtree rooted at 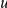
's child is created, one copy is attached as child of one of 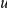
's original parents, and the other is attached as a child of 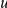
's other original parent. For example, in Figure 1, traversing the phylogenetic network from the leaves towards the root, the reticulation node 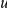 is encountered, two copies of the subtree rooted at its child (i.e., the most recent common ancestor of 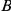 and 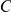) are created, and one is attached as a child of 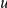
's parent 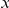, and the other is attached as a child of 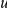
's parent 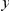, resulting in the MUL tree shown in the figure. In order to keep track of which branches in the MUL tree originated from the same branch in the phylogenetic network, we build during the conversion a mapping 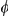 from the set of the MUL tree branches to the set of the phylogenetic network branches, such that 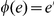 if branch 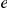 in the MUL tree corresponds to branch 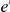 in the phylogenetic network. We make use of 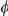 in two ways. The first is in transferring the branch lengths and hybridization probabilities from 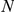 to the resulting MUL tree 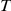, as illustrated briefly in Figure 1 and in more details in Text S1, and the second use is for computing the probabilities of gene trees, as becomes clearer below. Upon completion of this step of converting the phylogenetic network 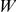, its branch lengths 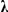 and hybridization probabilities 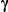, the result is a MUL tree 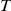 along with its branch lengths 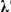, hybridization probabilities 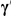, and the branch mapping 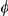. The full description of the procedure NetworkToMULTree for achieving this conversion is given in Text S1.

#### Step 2: Mapping the alleles to the leaves of the MUL tree

In computing the probability of a gene tree given a species phylogeny (tree or network), all the alleles sampled from species 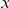 are mapped to the single leaf labeled 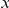 in the species phylogeny. However, unless the species phylogeny 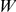 does not have any reticulation nodes, the resulting MUL tree 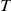 contains leaf sets that are labeled by the same species 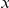. For example, in Figure 1, the MUL tree has two leaves labeled 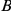 and two leaves labeled 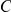. In this case, it is important to map the alleles systematically to the leaves of the MUL tree so as to cover *exactly* all the coalescence patterns that would arise had the alleles been mapped to the phylogenetic network.

We denote by 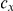 the set of leaf nodes in 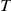 that are labeled by species 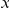. For example, 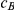 for the MUL tree in Figure 1 is the set of the two leaves labeled by 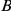. Now, consider a locus 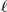. We denote by 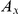 (for 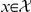) the set of alleles sampled from species 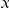 for locus 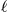, and by 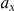 the size of this set (i.e., 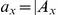). In the example of Figure 1, two alleles were sampled from species 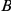; hence, 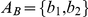 and 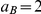. A *valid allele mapping* is a function 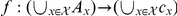 such that if 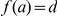, and 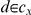, then 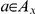. In other words, 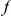 maps an allele from species 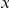 to a leaf in the MUL tree labeled by 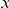. Let 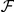 denote the set of all such valid allele mappings 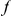; in Figure 1, 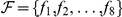.

#### Step 3: Computing the probability of a gene tree on the MUL tree

Once the phylogenetic network 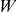 is converted into MUL tree 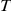 and the set of all valid allele mappings is produced (a straightforward computational task, yet results in a number of valid allele mappings that is exponential in a combination of the number of alleles sampled and the number of reticulation nodes), the probability of observing gene tree topology 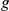 is found by summing the probability of 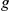 given the MUL tree over all possible allele mappings. Then, the probability of observing gene tree topology 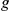 is found by summing over all possible allele mappings:
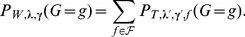
(3)In this equation, the 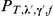 term accounts for all coalescent histories of a given mapping, which, when combined with the summation over all valid allele mappings, accounts for all coalescent histories within the branches of a phylogenetic network. Finally, the likelihood for a collection of gene trees is the product of the individual gene tree probabilities. This formulation naturally gives rise to a likelihood setup for estimating the parameters of a reticulate evolutionary history from a collection of gene trees described by their topologies.

To complete our framework, we now provide a formula for 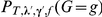, which is the probability of a gene tree given a MUL tree and a valid allele mapping. Special attention needs to be paid to sets of branches in the MUL tree that correspond to single branches in the phylogenetic network, since coalescence events within these branches are not independent. Let us illustrate this issue using valid allele mapping 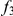 and the MUL tree 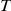 in Figure 1. Under this mapping, each of the two alleles sampled from species B is mapped to a different B leaf in 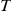. Tracing these two alleles independently from the two B leaves implicitly indicates that tracing the evolution of these two alleles in the phylogenetic network, no coalescence event should occur within time 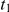 on the branch incident into leaf B in the network. Additionally, each branch in the MUL tree may have a hybridization probability associated with it that is neither 0 nor 1, and must be accounted for in computing the probabilities. Accounting for these two cases gives rise to
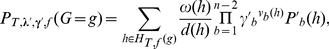
(4)where the 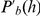 terms are symbolic quantities, that do not individually evaluate to any value. Instead, they play a role in simultaneously computing the probability along pairs of branches in the MUL tree that share a single source branch in the phylogenetic network. More formally, let 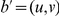 be a branch in 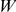 such that 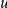 is a reticulation node. Given the mapping 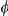 from the branches of 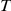 to the branches of 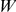, the pre-image (or, inverse image) 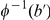 is the set of all branches in 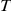 that map to 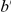 under 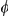. That is, 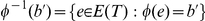, where 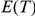 is the set of 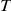
's branches. Then, we define
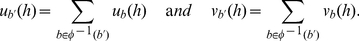
(5)This equation states that the number of lineages 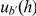 that enters (working backward in time) branch 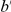 in the phylogenetic network equals the sum of the numbers of lineages that enter all branches of the MUL tree that map to branch 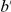. The number of lineages 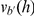 that exists branch 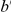 is defined similarly. In Figure 1, the number of lineages that enters branch 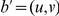 in the phylogenetic network equals the sum of the number of lineages that enter branch 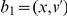 and the number of lineages that enter branch 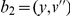 in the MUL tree.

Then, we use the following equation to evaluate the probability in Equation (4):
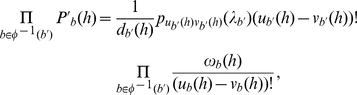
(6)where 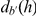 is computed using the formula in [26], with 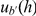 and 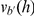 as parameters. In the example of branches 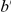, 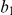 and 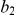 that we just illustrated, Equation (6) states that 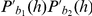 evaluates to
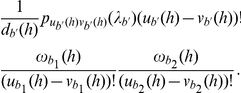
The term 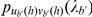 gives the probability that 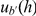 lineages coalesce into 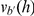 lineages within time 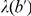. The term
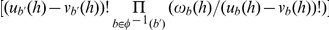
corresponds to the quantity 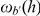 in [26]. Finally, the term
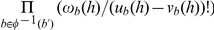
is the number of restrictions for the ordering of coalescent events within branch 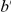.

### Accounting for uncertainty in gene tree topologies

Thus far, we have assumed that we have an accurate, fully resolved gene tree for each locus. However, in practice, gene tree topologies are inferred from sequence data and, as such, there is uncertainty about them. In Bayesian inference, this uncertainty is reflected by a posterior distribution of gene tree topologies. In a parsimony analysis, several equally optimal trees are computed. We propose here a way for incorporating this uncertainty into the framework above. Assume we have 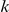 loci under analysis, and for each locus 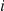, a Bayesian analysis of the sequence alignment returns a set of gene trees 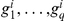, along with their associated posterior probabilities 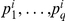 (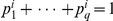). Now, let 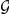 be the set of all distinct tree topologies computed on all 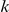 loci, and for each 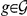 let 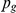 be the sum of posterior probabilities associated with all gene trees computed over all loci whose topology is 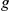. Thus, 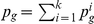 and 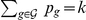. Then, we replace Eq. (1) by
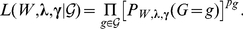
(7)We note that if 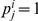 or 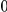 for each 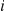 and 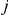, then Eq. (7) is equivalent to Eq. (1), and both are multinomial likelihoods. This multinomial approach has also been used elsewhere for both species networks under simple hybridization scenarios [28] and species trees [24]. We additionally allow the 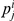 terms to be between 0 and 1 (and therefore 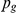 to be non-integer values) in order to reflect uncertainty in the estimated gene trees.

In the case where a maximum parsimony analysis is conducted to infer gene trees on the individual loci, a different treatment is necessary, since for each locus, all inferred trees are equally optimal. For locus 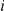, let 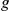 be the strict consensus of all optimal gene tree topologies found. Then, Eq. (1) becomes
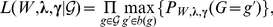
(8)where 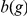 is the set of all binary refinements of gene tree topology 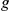.

## Results

### Support for hybridization in yeast

Using our method to compute the likelihood function given by Eq. (1), we reanalyzed the yeast data set of [35], which consists of 106 loci, each with a single allele sampled from seven Saccharomyces species *S. cerevisiae* (*Scer*), *S. paradoxus* (*Spar*), *S. mikatae* (*Smik*), *S. kudriavzevii* (*Skud*), *S. bayanus* (*Sbay*), *S. castellii* (*Scas*), *S. kluyveri* (*Sklu*), and the outgroup fungus *Candida albicans* (*Calb*). Given that there is no indication of coalescences deeper than the MRCA of *Scer*, *Spar*, *Smik*, *Skud*, and *Sbay* [36], we focused only on the evolutionary history of these five species (see Text S1). We inferred gene trees using Bayesian inference in MrBayes [37] and using maximum parsimony in PAUP* [38] (see Text S1 for settings).

**Figure 2:**
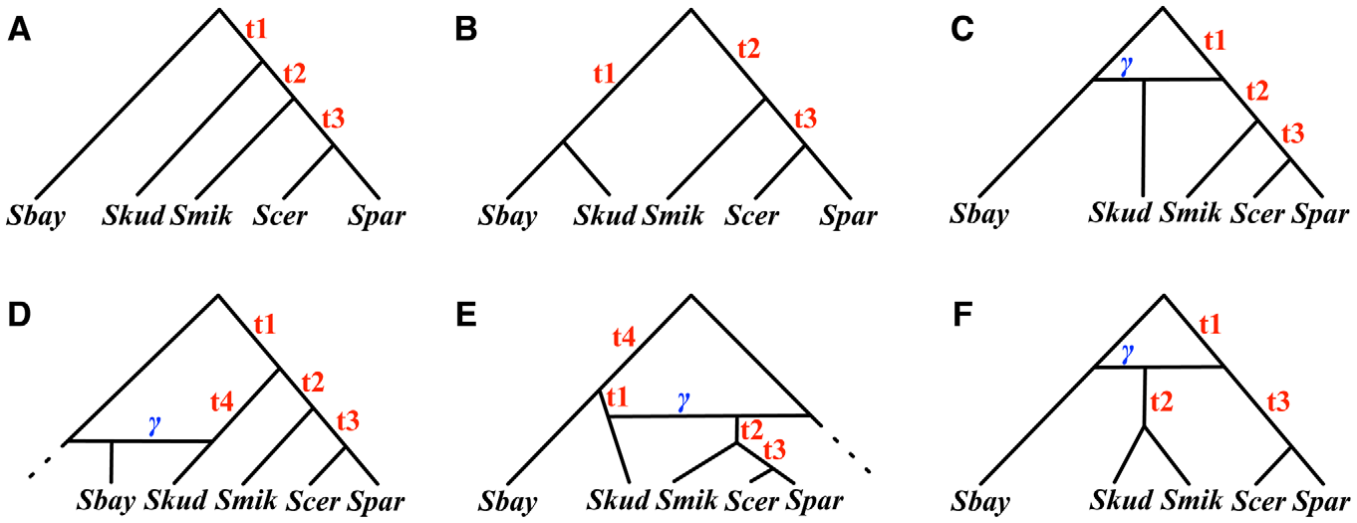
Various hypotheses for the evolutionary history of a yeast data set. (A) The species tree for the five species *Sbay*, *Skud*, *Smik*, *Scer*, and *Spar*, as proposed in [35], and inferred using a Bayesian approach [39] and a parsimony approach [36]. (B) A slightly suboptimal tree for the five species, as identified in [36], [39]. (C–E) The three phylogenetic networks that reconcile both trees in (A) and (B), and which we reported as equally optimal evolutionary histories under a parsimony criterion in [30]. (F) A phylogenetic network that postulates *Smik* and *Skud* as two sister taxa whose divergence followed a hybridization event.

The species tree that has been reported for these five species, based on the 106 loci, is shown in Figure 2A [35]. Further, additional studies inferred the tree in Figure 2B as a very close candidate for giving rise to the 106 gene trees, under the coalescent model [36], [39]. Notice that the difference between the two trees is the placement of *Skud*, which flags hybridization as a possibility. Indeed, the phylogenetic network topologies in Figure 2C and 2D have been proposed as an alternative evolutionary history, under the stochastic framework of [40], as well as the parsimony framework of [30].

Using the 106 gene trees, we estimated the times 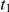, 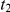, 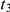, 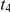 and 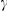 for the six phylogenies in Figure 2 that maximize the likelihood function (we used a grid search of values between 0.05 and 4, with step length of 0.05 for branch lengths, and values between 0 and 1 with step length of 0.01 for 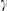). Table 1 lists the values of the parameters computed using Eq. (7) on the gene trees inferred by MrBayes and Table 2 lists the values of the parameters computed using Eq. (8) on the gene trees inferred by PAUP*, as well as the values of three information criteria, AIC [31], AICc [32] and BIC [33], in order to account for the number of parameters and allow for model selection.

**Table 1:** Analysis results for the six phylogenies in Figure 2 using gene tree topologies inferred by a Bayesian analysis (using MrBayes).

Species phylogeny	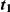	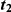	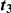	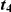	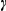	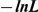	AIC	AICc	BIC
Figure 2A	0.05	0.85	2.05	N/A	N/A	284	575	576	583
Figure 2B	0.2	0.85	2.05	N/A	N/A	276	559	560	567
Figure 2C	0.4	0.65	2.05	N/A	0.59	274	556	556	567
Figure 2D	2.95	0.7	2.1	0.85	0.5	247	504	504	517
Figure 2E	0.6	0.05	2.05	0.2	0.0	276	563	564	577
Figure 2F	0.9	0.05	2.15	N/A	0.27	325	659	659	669

**Table 2:** Analysis results for the six phylogenies in Figure 2 using gene tree topologies inferred by maximum parsimony (using PAUP*).

Species phylogeny	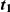	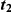	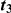	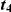	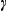	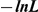	AIC	AICc	BIC
Figure 2A	0.3	1.25	3.6	N/A	N/A	205	416	417	424
Figure 2B	0.2	1.35	3.6	N/A	N/A	208	423	423	431
Figure 2C	1.1	1.05	3.6	N/A	0.34	188	384	385	395
Figure 2D	3.45	1.15	3.6	3.05	0.34	157	325	326	338
Figure 2E	0.3	1.25	3.6	N/A	1.0	205	420	421	434
Figure 2F	1.55	0.05	3.7	N/A	0.18	252	512	512	523

Out of the 106 gene trees (using either of the two inference methods), roughly 100 trees placed *Scer* and *Spar* as sister taxa, which potentially reflects the lack of deep coalescence involving this clade (and is reflected by the relatively large 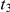 values estimated). Roughly 25% of the gene trees did not show monophyly of the group *Scer*, *Spar*, and *Smik*, thus indicating a mild level of deep coalescence involving these three species (and reflected by the relatively small 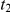 values estimated). However, a large proportion of the 106 gene trees indicated incongruence involving *Skud*; see . This pattern is reflected by the very low estimates of the time 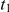 on the two phylogenetic trees in Figure 2. On the other hand, analysis under the phylogenetic network models of Figure 2C and 2D indicates a larger divergence time, with substantial extent of hybridization. These latter hypotheses naturally result in a better likelihood score. When accounting for model complexity, all three information criteria indicated that these two phylogenetic network models with extensive hybridization and larger divergence time between *Sbay* and the ( *Smik*,( *Scer,Spar*)) clade provide better fit for the data. Further, while both networks produced identical hybridization probabilities, the network in Figure 2D had much lower values of the information criteria than those of the network in Figure 2E. The networks in Figure 2E and 2F have lower support (under all measures) than the other four phylogenies. In summary, our analysis gives higher support for the hypothesis of extensive hybridization, a low degree of deep coalescence, and long branch lengths than to the hypothesis of a species tree with short branches and extensive deep coalescence. It is worth mentioning that while the three networks in Figure 2C–2E were reported as equally optimal under a parsimonious reconciliation [36], our new framework can distinguish among the three, and identifies the network in Figure 2D as best, followed by the one in Figure 2C (the network of Figure 2E is found to be a worse fit than either of the two species tree candidates).

### Support for hybridization in *Drosophila*

We reanalyzed the three-species *Drosophila* data set of [41], which includes *D. melanogaster* ( *Dmel*), *D. yakuba* ( *Dyak*), and *D. erecta* ( *Dere*).

The data set consisted of 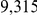 loci supporting the three possible gene tree topologies as follows:

- gene tree 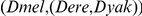 is supported by 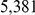 (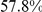) loci;
- gene tree 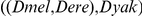 is supported by 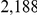 (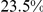) loci; and,
- gene tree 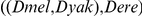 is supported by 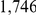 (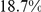) loci.

For a species tree with three species and one individual sampled per species, the multispecies coalescent predicts that the two gene trees with topologies different from that of the species tree each occur with probability 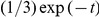, where 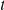 is the length of the one internal branch in coalescent units [42]. Two important predictions under the coalescent are therefore that the two nonmatching gene trees are expected to be tied in frequency and that both occur less than 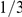 of the time, with the matching gene tree topology occurring more than 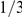 of the time. This tie in the expected frequency of nonmatching gene trees is observed in some three-taxon data sets, but not in others, including the *Drosophila* data set.

**Figure 3:**
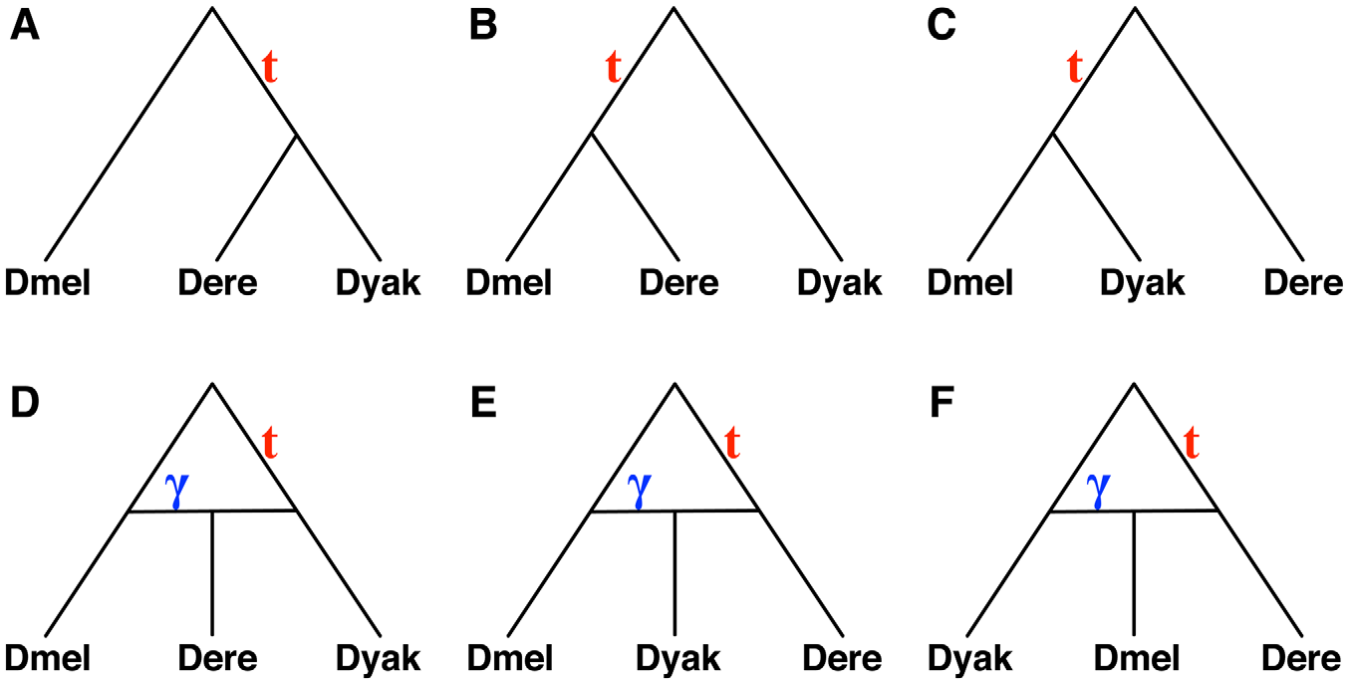
Six hypotheses for the evolutionary history of a *Drosophila* data set. (A–C) The three possible species tree topologies. (D–E) The three possible single-hybridization species network topologies (excluding extinction events).

Although this deviation from symmetry can be explained by a model of population subdivision, where the subdivision must occur in the internal branch as well as the population ancestral to all three species [43], the asymmetry can also be explained by the simplest hybridization network on three species with just one hybridization parameter (Figure 3).

We considered six candidates for the species phylogeny: three with no hybridization, and three with hybridizations involving different pairs of species (see Figure 3). For the three phylogenetic trees, we estimated the time 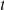 that maximizes the probability of observing all 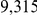 gene trees, and for the three phylogenetic networks, we additionally estimated the hybridization probability 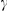.

The results in Table 3 show that of the three phylogenetic trees, the one in Figure 3A provides the best fit of the data, which is in agreement with the analysis in [41]. In fact, the value of 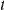 we estimated on the other two trees was the lowest value we used in the estimation procedure. Clearly, this value can be arbitrarily small for these two trees, since the unresolved phylogeny ( *Dmel*, *Dere*, *Dyak*) fits the data better.

**Table 3:** Estimates of time 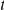 and hybridization probability 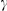 (when applicable) on the six candidate species phylogenies shown in Figure 3 for the three *Drosophila* species *Dmel*, *Dere*, and *Dyak*.

Species phylogeny	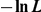	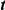	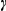	AIC	AICc	BIC
Figure 3A	9070	0.46	N/A	18143	18143	18150
Figure 3B	10233	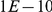	N/A	20469	20469	20476
Figure 3C	10233	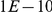	N/A	20469	20469	20476
Figure 3D	9045	0.58	0.11	18095	18095	18109
Figure 3E	9070	0.46	0.0	18145	18145	18159
Figure 3F	10233	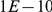	0.0	20471	20471	20485

Among the three network candidates, the one in Figure 3D has the best fit of the data. This network, with a value of 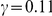, indicates that 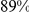 of the alleles sampled from *Dere* shared a common ancestor first with alleles from *Dyak* (reflecting the tree in Figure 3A), while 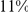 of the alleles from *Dere* shared a common ancestor first with alleles from *Dmel* (reflecting the tree in Figure 3B). Indeed, this network is the smallest network (in terms of the number of reticulation nodes) that reconciles both trees. Further, the change in AIC for this network is 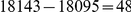, indicating a much better fit than the best tree (Figure 3A). As noted previously [43], a 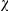-square test will also strongly reject the hypothesis that the species relationships are tree-like with random mating.

This three-taxon example can be analyzed analytically. Fitting a hybridization parameter allows a perfect fit to any observed frequencies of gene tree topologies for three species for one of the three networks in Figure 3. We let 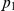, 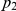, and 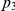 represent the probabilities of topologies (*Dmel*,( *Dere*, *Dyak*)), ((*Dmel*, *Dere*), *Dyak*), and ((*Dmel*, *Dyak*), *Dere*) under the network in Figure 3D. Then
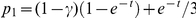


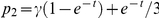


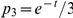



This system has the unique solution
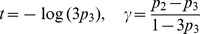
(9)for 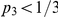 and 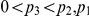 (either at least one of the gene tree probabilities is less than 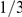 if since they sum to 1.0; or if they are all exactly 1/3, then a star tree with 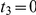 and any 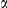 exactly fits the data). Thus we can estimate 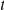 and 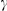 using the observed 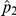 and 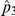 in equation (9), and this also maximizes the likelihood.

### Identifiability of hybridization using gene tree topologies: A simulation study

For the simulated data, we evolved gene trees within the branches of phylogenetic networks, while varying branch lengths and hybridization probabilities, and investigated two questions: (1) how much data (gene trees) is needed to obtain accurate inference of the parameters (branch lengths and/or hybridization probabilities)? (2) are the parameters always identifiable? To answer these two questions, we investigated six different phylogenetic network topologies that involved single reticulation scenario, two reticulation scenarios (dependent and independent), and cases with extinctions involving the species that hybridize (see Text S1).

Our results show that both hybridization probabilities and branch lengths can be estimated with very high accuracy provided that no extinction events were involved in the parents of hybrid populations (see Text S1). Further, this accuracy can be achieved even when using the smallest number of gene trees we used in our study, which is 10. Under these settings, estimates using our framework seemed to converge quickly to the true values.

**Figure 4:**
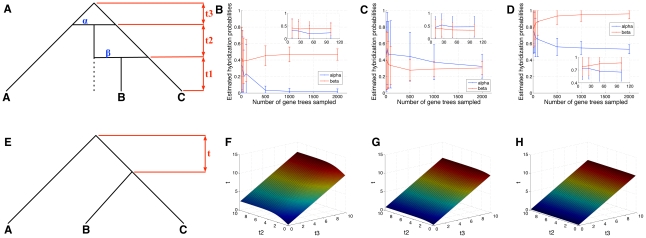
Identifiability in detecting hybridization. (A) A phylogenetic network with two hybridization probabilities, where the second hybridization involves the first hybrid population, and extinction is involved. (B–D) Estimates of 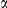 and 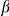, as a function of the number of gene trees used, when the true values of 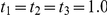 are assumed in the inference, and for true 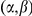 values of 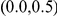, 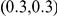, and 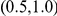, respectively (insets zoom in on the left parts of the figure). (E) A phylogenetic tree with three taxa, and with divergence time 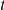 between the two speciation events. (F–H) The value of 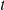 for the tree in (E) that yields the same probability of the data under the scenario depicted in (A) when 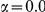, as a function of 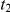 and 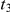, and for 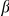 value of 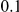, 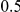, and 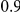, respectively. Since a single allele was sampled per species, the data is uninformative for estimating the value of 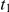 here.

We also investigated the performance of the method, as well as identifiability issues when phylogenetic signal from at least one of the species involved in the hybridization is completely lost. Figure 4 shows the results for one such scenario (see Text S1 for another scenario that involves the loss of phylogenetic signal from both species involved in the hybridization).

Panels Figure 4B–4D show that when the true values of 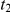 and 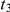 are assumed to be known in the estimation procedure (the value of 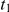 is irrelevant in the case when a single allele is sampled per species), the estimates of the hybridization probabilities converge to the true values. However, unlike the cases that did not involved extinctions, a larger number of gene trees is now required to obtain an accurate estimate (while there are only three possible gene tree topologies, a large number of gene trees need be sampled in order for the three topologies' frequencies to be informative). The time intervals of 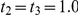 coalescent units amount to a large extent of deep coalescence events, which blurs the phylogenetic signal, and results in slight over- or under-estimation of the hybridization probabilities (Text S1 shows the results for the time interval with 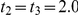).

If the topology of the network in Figure 4A is assumed to be known, but both the branch lengths and hybridization probabilities are to be estimated, then these parameters are unidentifiable; that is, two different pairs of vectors of branch lengths and hybridization probabilities can be found to explain the observed data with exactly the same probability (see Text S1). If at least two alleles are sampled from species B, then the parameter values become identifiable; however, an extremely large, and potentially infeasible, number of gene trees need to be sampled to uniquely identify the parameter values in practice (see Text S1).

Furthermore, in the special case where 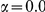, a phylogenetic tree, with appropriate branch lengths can be found, to fit the data exactly with the same probability that the phylogenetic network would. Let 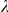 be the branch lengths vector with 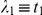, 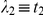, and 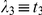, and let 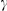 be the hybridization probabilities vector with 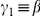. Now, consider the phylogenetic tree 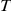 in Figure 4E. Then, if we set 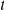 as a function of 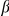, 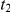, and 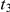, using 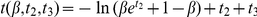, then, 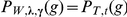 for any gene tree 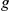. The values of 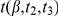 are shown in Figure 4F–4H. These results show that as 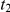 increases, the value of 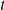 becomes unaffected by 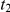, and that increasing 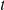 proportionally to the increase in 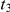 always maintains identical probabilities of gene trees under both species phylogenies (see Text S1).

Our method for computing the probability of gene trees under hybridization and deep coalescence allows for analyzing data sets with arbitrary complexity of evolutionary histories in terms of the hybridization scenarios. When parameters are identifiable, our method estimates their values with high accuracy from a relatively small number of loci. Further, our method can be used to show lack of identifiability of model parameters for other cases. Our method supports a hypothesis of larger divergence time coupled with hybridization over short divergence times (with extensive deep coalescence) in a yeast data set. Finally, for a large *Drosophila* data set, our method indicated no hybridization based on the sampled loci.

## Discussion

### Using coalescence times versus topologies to infer species networks

We have focused on calculating probabilities of gene tree topologies and using these probabilities to infer species networks. In addition, the joint density of the coalescence times and topology in the gene trees could be used to infer species networks. Indeed, this approach has been used for networks where reticulation nodes have one descendant which is an extant species [29], using the density for coalescence times derived by Rannala and Yang [44]. This approach is computationally faster than computing gene tree topology probabilities because it is not necessary to sum over a large number of coalescent histories. To compute this joint density, each gene sampled can potentially have to trace through up to 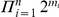 possible paths through the network, where 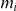 is the number of hybridization events ancestral to the sampled gene from species 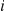, and the density will take the form of a sum over possible paths through the network. (In contrast, computing the probability of a topology will require 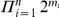 mappings of alleles to the MUL-tree, and each gene topology calculation will require summing over coalescent histories.) This joint density for the gene trees with coalescence times could then be used in either maximum likelihood or Bayesian frameworks to infer the species network.

An important advantage of using coalescence times is that certain networks might be identifiable using coalescence times when probabilities of topologies might not identify the network. In the example of Figure 3A, although the gene tree topology probabilities can be obtained by a tree, the distribution of the coalescence times between lineages sampled from B and C is a mixture of three shifted exponential distributions if 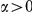, but a mixture of two shifted exponential distributions if 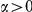. For example, if 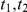, and 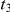 are known but 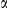 and 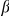 are unkown, then the likelihood of observing a coalescence between a B and C lineage for times slightly greater 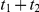 will be very low if 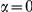, and much higher for 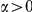, thus making it possible to test whether 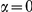 when coalescence times are used.

Another identifiability issue is that both population subdivision and hybridization can lead to the asymmetry in gene tree topology probabilities in the 3-taxon case such as observed in the *Drosophila* example discussed earlier, where the two least frequently observed topologies are not tied in frequency. Either population subdivision, with a parameter describing the probability that the two most closely related species fail to coalesce in the ancestral population due to population structure, or hybridization can fit the data for the gene tree topologies. However, the two models could imply different distributions on coalescence times, which might therefore be useful in distinguishing the models. We note that identifiability in the case of three species with one individual per species might be especially limited due to the small number of gene tree topology probabilities that can be used to estimate parameters. In the case of identifying rooted species trees from unrooted gene trees with one lineage per species, for example, identifiability is achieved only with 5 or more species [17].

We consider it desirable to develop many methods for inferring species trees and species networks so that their properties and performances can be compared. In the case of species tree inference, there are advantages and disadvantages to using topology-based methods versus methods that include branch lengths, and in using likelihood versus Bayesian methods. We expect that many of these strengths and weaknesses may carry over to the case of inferring networks. For moderately sized data sets, Bayesian methods that model branch lengths and uncertainty in the gene trees such as BEST [45] and *BEAST [46] often have the best performance [47]. However, these methods require estimating the joint posterior distribution of the species tree and gene trees and therefore are difficult to implement for large numbers of loci. Maximizing the likelihood of the gene trees and their coalescent times (but without accounting for uncertainty in the gene trees), as in STEM [48], is fast and has very good performance on known gene trees but seems to be very sensitive to the assumption that branch lengths are estimated correctly [24], [49]. Maximizing the likelihood of the species tree using only gene tree topologies using the program STELLS, even while not accounting for uncertainty in the gene trees, tended to have better performance than STEM for a large simulated data set (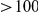 loci on 8 taxa) and worse performance on fewer loci [24]. Which method is optimal for inferring species trees or networks might depend on many factors such as the number of loci, the number of lineages sampled per species, the accuracy with which branch lengths can be estimated, the extent to which there are model violations, and the speciation history [49].

### Recombination and population size assumptions

Two common assumptions in multispecies coalescent models are that there is no recombination within loci (and free recombination between loci) and that ancestral population sizes are constant.

Recombination can lead to different portions of a gene alignment effectively having distinct gene tree topologies. Ideally, alignments should be chosen so that recombination within genes is unlikely. This can be achieved by testing alignments beforehand for recombination using many available methods [50]–[52], or for whole genome data, choosing the cutoffs for loci such that they are unlikely to occur at recombination breakpoints [53]. In addition, recombination may lead to greater violations of the coalescent model for branch lengths than for topologies [53], so that topology-based methods might be less sensitive to the assumption that there is no recombination within loci. In addition, a recent simulation study found that recombination within loci did not have much impact on species tree inference methods for a wide range of recombination rates [54].

Coalescent models often assume that ancestral populations have constant size for the duration of the population (i.e., a constant size for a given branch of the species tree, but not necessarily the same on different branches). The program *BEAST [46] allows for ancestral population sizes to change linearly with time. Nonconstant population sizes will tend to result in branch lengths that make topologies more (or less) star-like for populations that are increasing (or decreasing) in size [55]. One approach to modelling a changing population size would be to break up a branch into intervals that are relatively constant in size. Suppose, for instance that a branch consists of an interval of 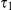 generations with population size 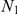, and 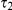 generations with size 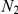. The total time of the branch in coalescent units is 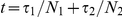. Although unequal values of 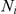 can affect the distribution of coalescence times (for example, if 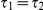 but 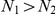, then coalescence events might be more likely to occur in the interval with size 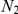), the probabilities of topologies arising in this branch are not affected and can be calculated just using the total time 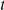. In particular, for the functions 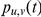, which are the terms that depend on time in the calculations for gene tree topology probabilities, we have
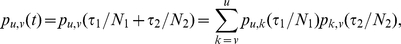
which is an instance of the Chapman-Kolmogorov equations because the number of lineages is a continuous time Markov chain (a death chain) [56].

We expect that topology-based methods may show more robustness to recombination and changing population sizes than approaches which explicitly model coalescence times. However, for estimating species trees and networks from gene trees, as in other areas of statistical inference, there is likely to be a tradeoff between power and robustness for methods that do and do not model branch lengths of the gene trees.

### Searching for networks

A current limitation to the procedure we have outlined for estimating hybridization is that we require a set of candidate networks on which to perform model selection. In some cases, such a set of candidate networks can be obtained by considering specific hypotheses related to biogeographical information. Candidate networks can also be generated using supernetworks from gene trees [57] or other network methods [9]. Often these methods will generate very complicated networks if there are many conflicts in the data, so it might be useful to choose different random subsets of well-supported (or frequently occurring) gene tree topologies to generate candidate species networks. In the future it will be desirable to develop algorithms that directly search the space of species networks in order to automate searching for optimal species networks.

## Supporting Information

Text S1 Supporting information file that contains formal definitions and additional results on synthetic data.
(PDF)
