# Supplementary material for: The Probability of a Gene Tree Topology within a Phylogenetic Network with Applications to Hybridization Detection
Source: PLoS Genet. 2012 Apr 19;8(4):e1002660. doi: 10.1371/journal.pgen.1002660 (PMC3330115; doi:10.1371/journal.pgen.1002660)
Supplement: Text S1 — Supporting information file that contains formal definitions and additional results on synthetic data. (PDF) [file pgen.1002660.s001.pdf]

Supplementary Material:
The Probability of a Gene Tree Topology Within a Phylogenetic Network With Applications to Hybridization Detection

Yun Yu[1], James H. Degnan[2], Luay Nakhleh[1,*]
1 Computer Science, Rice University, Houston, Texas, USA
2 Mathematics and Statistics, University of Canterbury, Christchurch, New Zealand
∗ E-mail: nakhleh@cs.rice.edu

Contents

1 Phylogenetic networks **2**

2 From a phylogenetic network to a Multilabeled (MUL) tree **3**

3 Coalescent histories on phylogenetic networks and their MUL trees **5**

4 The yeast data set **7**

5 Simulating gene genealogies **10**

6 Accuracy of inference **12**

7 Identifiability **17**

8 References **23**

1 Phylogenetic networks

The term *phylogenetic network* has grown to become an umbrella term that encompasses any non-treelike model [1]; therefore, it is important to explicitly describe the phylogenetic network model used. Since we are concerned with hybridization and deep coalescence, we use the evolutionary, or hybridization, phylogenetic network model given in [2], which we now briefly review.

Definition 1 *A* phylogenetic $\mathcal{X}$-network*, or $\mathcal{X}$-network for short, W is an ordered pair (G, ℓ), where $G = (V, E)$ is a directed, acyclic graph (DAG) with $V = \{r\} \cup V_L \cup V_T \cup V_N$, where*

- *$indeg(r) = 0$ (r is the* root *of W);*
- *$\forall v \in V_L$, $indeg(v) = 1$ and $outdeg(v) = 0$ (V_L are the* external tree nodes*, or* leaves*, of W);*
- *$\forall v \in V_T$, $indeg(v) = 1$ and $outdeg(v) \geq 2$ (V_T are the* internal tree nodes *of W); and,*
- *$\forall v \in V_N$, $indeg(v) = 2$ and $outdeg(v) = 1$ (V_N are the* reticulation nodes *of W),*

$E \subseteq V \times V$ are the network's edges (we distinguish between reticulation edges*, edges whose heads are reticulation nodes, and* tree edges*, edges whose heads are tree nodes), and $\ell : V_L \rightarrow \mathcal{X}$ is the* leaf-labeling *function, which is a bijection from V_L to $\mathcal{X}$.*

We use $V(W)$ and $E(W)$ to denote the set of nodes and edges of phylogenetic network W. Fig. S1 shows an example of a phylogenetic network based on Definition 1.

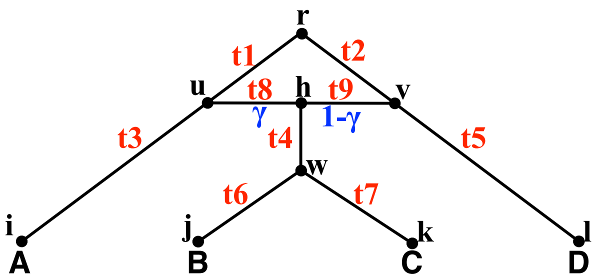

Fig. S1. A phylogenetic network W, and its associated branch lengths and hybridization probabilities. The network has 9 nodes (solid circles), which include the root r, one reticulation node, h, 4 leaves (bijectively labeled by the set $\mathcal{X} = \{A, B, C, D\}$), and 3 internal tree nodes. Shown also are the branch lengths (red) and hybridization probabilities (blue).

In addition to the topology of a phylogenetic network W, we associate with each branch $b = (u, v)$ in the network a branch length, denoted by λ_b (equivalently, $\lambda_{(u,v)}$), which reflects the time in coalescent units between the two endpoints of the branch. To describe all branch lengths of a phylogenetic network, a vector $\boldsymbol{\lambda}$ with one entry per branch is provided. In addition, for each reticulation node h, with two parent edges $b_1 = (u, h)$ and $b_2 = (v, h)$, we associate hybridization probabilities γ_{b_1} (equivalently, $\gamma_{(u,h)}$) and γ_{b_2} (equivalently, $\gamma_{(v,h)}$), such that $\gamma_{b_1} + \gamma_{b_2} = 1$. The parameter $\gamma_{(x,h)}$ is taken to denote the proportion of alleles in the population h that are inherited from population x. To describe all hybridization probabilities associated with a phylogenetic network, a vector $\boldsymbol{\gamma}$ with one entry per reticulation edge is provided.

2 From a phylogenetic network to a Multilabeled (MUL) tree

Central to our formulation/algorithm for computing the probability of a gene tree given a phylogenetic network is converting the phylogenetic network to a multilabeled tree, or MUL tree [3]. A MUL tree is not a true phylogenetic tree, since its leaves are not uniquely labeled by a taxa set. However, we show in this work that the MUL tree representation of a phylogenetic network allows us to extend coalescent-based calculations of gene tree probabilities in a straightforward manner to cases where hybridization may be involved.

It is straightforward to convert a phylogenetic network into its corresponding MUL tree. The main idea is to process the phylogenetic network in a bottom-up fashion, traversing its nodes from the leaves towards the root. Every time a reticulation node h is encountered, two copies of the tree rooted at its child w are created, and each of h's two parents points to exactly one of the two copies. As the traversal operates in a bottom-up fashion, it is guaranteed that when a reticulation node is encountered, there are no reticulation nodes remaining "under" it (they would have been processed already). In addition to the topology, the conversion maps the branch lengths and hybridization probabilities to the appropriate branches as well. Finally, as a single edge in a phylogenetic network W may give rise to multiple edges in the MUL tree T, Algorithm **NetworkToMULTree** returns a mapping $\phi : E(T) \to E(W)$ that keeps track of this information.

The MUL tree T that corresponds to the phylogenetic network W of Fig. S1 is given in Fig. S2. In this example, we have the following values of $\phi : E(T) \to E(W)$:

- $\phi((u,i)) = (u,i)$, $\phi((v,l)) = (v,l)$, $\phi((r,u)) = (r,u)$, and $\phi((r,v)) = (r,v)$.
- $\phi((u,w)) = \phi((v,w')) = (h,w)$.
- $\phi((w,j_1)) = \phi((w',j_2)) = (w,j)$.
- $\phi((w,k_1)) = \phi((w',k_2)) = (w,k)$.

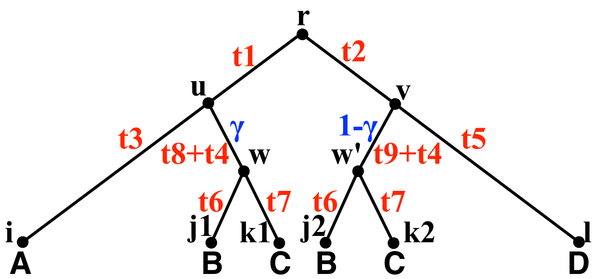

Fig. S2. The MUL tree, branch lengths (red), and hybridization probabilities (blue), that correspond to the phylogenetic network of Fig. S1, as generated by Algorithm 1. In the MUL tree, each branch has a hybridization probability; values not shown here equal 1.

The conversion procedure is given formally in Algorithm 1 (**NetworkToMULTree**).

Algorithm 1: NetworkToMULTree.

Input: Phylogenetic $\mathcal{X}$-network W; branch lengths $\boldsymbol{\lambda}$; hybridization probabilities $\boldsymbol{\gamma}$.
Output: MUL tree T; branch lengths $\boldsymbol{\lambda'}$; hybridization probabilities $\boldsymbol{\gamma'}$; edge mapping $\phi : E(T) \rightarrow E(W)$.
$T \leftarrow W$ and set $\phi(e) = e'$ where $e \in E(T)$ is a copy of $e' \in E(W)$;
$\boldsymbol{\lambda'} \leftarrow \boldsymbol{\lambda}$;
foreach $b \in E(T)$ **do**
  $\gamma'_b \leftarrow 1$;
while *traversing the nodes of* T *bottom-up* **do**
  if *node* h *has two parents,* u *and* v*, and child* w **then**
    Create a copy of T_w whose root is new node w' and set $\phi(e) = e'$ where $e \in E(T_{w'})$ is a copy of $e' \in E(T_w)$;
    Add to T two new edges $e_1 = (u, w)$ and $e_2 = (v, w')$;
    $\phi_{e_1} \leftarrow (h, w)$; $\phi_{e_2} \leftarrow (h, w)$;
    $\lambda'_{(u,w)} \leftarrow \lambda_{(u,h)} + \lambda_{(h,w)}$; $\lambda'_{(v,w)} \leftarrow \lambda_{(v,h)} + \lambda_{(h,w)}$;
    $\gamma'_{(u,w)} \leftarrow \gamma_{(u,h)}$; $\gamma'_{(u,w)} \leftarrow \gamma_{(u,h)}$;
    Delete from T node h and edges (u, h), (v, h), and (h, w);
    Delete $\gamma'_{(u,h)}$, $\gamma'_{(v,h)}$, $\lambda'_{(u,h)}$, $\lambda'_{(v,h)}$, $\lambda'_{(h,w)}$, $\phi_{(u,h)}$, $\phi_{(v,h)}$, $\phi_{(h,w)}$;
return T;

It is important to note that it is possible that two different phylogenetic network topologies give rise to the same MUL-tree topology, and under certain settings of branch lengths and hybridization probabilities, the networks may also give rise to identical MUL-tree topologies and branch parameters (which, by definition, would result in non-identifiability of the topology and/or parameter values). However, if the parameter values differ between the two networks, they may still be identifiable, even though the two networks give rise to the same MUL-tree topology. This issue is illustrated in Fig. S3.

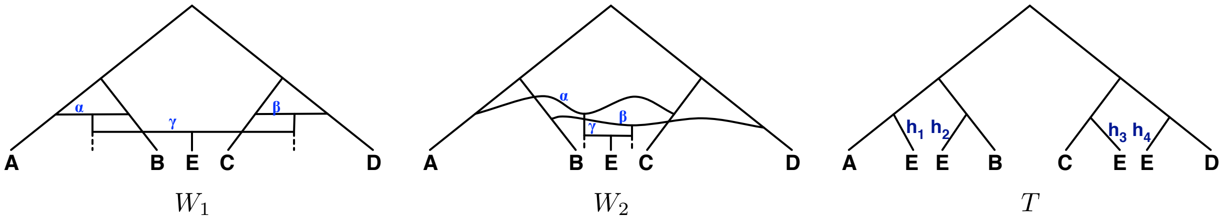

Fig. S3. Two phylogenetic networks N_1 and N_2 that give rise to the same MUL-tree toplogy.

The phylogenetic network W_1 involves a hybridization between A and B, a hybridization between C and D, and a hybridization of the two hybrids. The phylogenetic network W_2 involves a hybridization between A and C, a hybridization between B and D, and a hybridization between the two hybrids. When the MUL-tree T is obtained from W_1, then we have

- $h_1 = \alpha\gamma$, $h_2 = (1 - \alpha)\gamma$, $h_3 = \beta(1 - \gamma)$, and $h_4 = (1 - \beta)(1 - \gamma)$.

When the MUL-tree T is obtained from W_2, then we have

- $h_1 = \alpha\gamma$, $h_2 = \beta(1 - \gamma)$, $h_3 = (1 - \alpha)\gamma$, and $h_4 = (1 - \beta)(1 - \gamma)$.

Further, different lengths of the branches of the two networks would result in different branch lengths of the MUL-trees produced from each of the networks.

3 Coalescent histories on phylogenetic networks and their MUL trees

The notion of *coalescent histories* is central to computing gene tree probabilities [4]. Let $V(t)$ denote the set of nodes in a tree t, and let t_u denote the subtree of tree t that is rooted at node u. Given gene tree g and species tree T, a *coalescent history* is a function $h : V(g) \to V(T)$ such that the following conditions hold:

- if w is a leaf in g, then $h(w)$ is the leaf in T with the same label (in the case of multiple alleles, $h(w)$ is the leaf in T with the label of the species from which the allele labeling leaf w in g is sampled); and,
- if w is a node in g_v, then $h(w)$ is a node in $T_{h(v)}$.

Given a species tree T and a gene tree g, $H_T(g)$ denotes the set of all coalescent histories; mathematical properties and algorithms for computing $H_T(g)$ have been given [5,6].

A similar notion of coalescent histories can be defined on phylogenetic networks. Let W be a phylogenetic network and u be a node in $V(W)$. We denote by W_u the set of nodes in W that are under node u (that is, the set of nodes that are reachable from the root of W via at least one path that goes through node u). We can now define a coalescent history of a gene tree g and a species (phylogenetic) network W as a function $h : V(g) \to V(W)$ such that the following conditions hold:

- if w is a leaf in g, then $h(w)$ is the leaf in W with the same label (the same as above in the case of multiple alleles); and,
- if w is a node in g_v, then $h(w)$ is a node in $W_{h(v)}$.

The algorithm given in [6] for computing the set $H_T(g)$ does not apply to the case when the species phylogeny is a network; that is, for computing $H_W(g)$. Further, a phylogenetic network is parameterized with hybridization probabilities $\boldsymbol{\gamma}$ that must be associated properly with the coalescent histories to obtain the gene tree probability.

Let T be a MUL tree, g be a gene tree, and f be a valid allele mapping (see main text). Then, a *coalescent history* is a function $h : V(g) \to V(T)$ such that the following conditions hold:

- if w is a leaf in g, then $h(w) = f(a)$ where a is the allele that labels leaf w; and,
- if w is a node in g_v, then $h(w)$ is a node in $T_{h(v)}$.

We denote by $H_{T,f}(g)$ the set of all coalescent histories of gene tree g within the branches of MUL tree T given the valid allele mapping f.

Table S1 lists all the coalescent histories of the gene tree and MUL tree in Fig. S4. The allele mappings are given in Fig. 1 in the main text. Each row in the table gives the branches of the MUL tree on which the coalescent events, represented by the gene tree internal nodes x, y and z, occur.

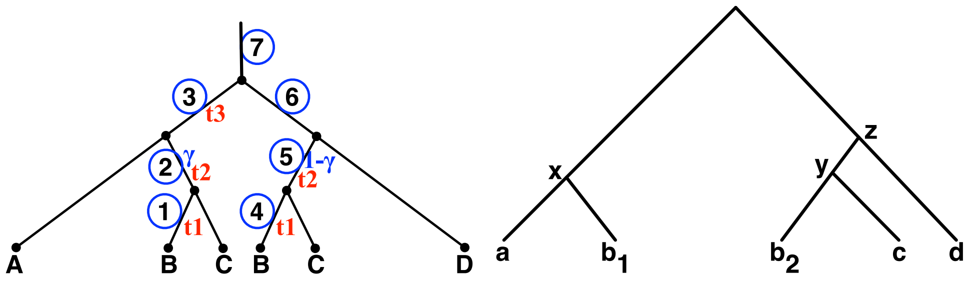

Fig. S4. (Left) The MUL tree from Fig. S2 with its branches numbered, and (Right) a gene tree with a single allele sampled from the three species A, C, and D, and two alleles sampled from species B.

Table S1. The coalescent histories of the gene tree topology and the MUL tree T of Fig. S4 (the valid allele mappings are given in Fig. 1 in the main text). x, y, and z are the internal nodes of the gene tree, and each number corresponds to the branch in the MUL tree to which the internal node of the gene tree is mapped.

Allele mapping	x	y	z
f_1	3	2	7
	3	3	7
	3	7	7
	7	2	7
	7	3	7
	7	7	7
f_2	3	7	7
	7	7	7
f_3	3	7	7
	7	7	7
f_4	3	5	6
	3	5	7
	3	6	6
	3	6	7
	3	7	7
	7	5	6
	7	5	7
	7	6	6
	7	6	7
	7	7	7
f_5	7	2	7
	7	3	7
	7	7	7
f_6	7	7	7
f_7	7	7	7
f_8	7	5	6
	7	5	7
	7	6	6
	7	6	7
	7	7	7

4 The yeast data set

We reanalyzed the yeast data set of [7], which consists of 106 loci, each with a single allele sampled from seven Saccharomyces species *S. cerevisiae* (*Scer*), *S. paradoxus* (*Spar*), *S. mikatae* (*Smik*), *S. kudriavzevii* (*Skud*), *S. bayanus* (*Sbay*), *S. castellii* (*Scas*), *S. kluyveri* (*Sklu*), and the outgroup fungus *Candida albicans* (*Calb*). Given that there is no indication of coalescences deeper than the MRCA of *Scer*, *Spar*, *Smik*, *Skud*, and *Sbay* [8], we focused only on the evolutionary history of these five species.

For our analysis, we reconstructed gene trees on all loci using Bayesian inference in MrBayes [9] and maximum parsimony in PAUP* [10].

For Bayesian inference, we used the GTR+Gamma+I model of sequence evolution, as well as the following setting of MCMC analysis, in MrBayes:

```
mcmc ngen=1000000 mcmcdiagn=yes relburnin=yes
burninfrac=0.25 stoprule=no stopval=0.01
```

For maximum parsimony, we used the following commands in PAUP* (when multiple optimal trees were found for a locus, we used the strict consensus of all of them):

```
set criterion=parsimony maxtrees=1000 increase=no;
outgroup Calb;
hs;
```

This step resulted in a set $\mathcal{G}$ of 106 gene trees, each of which was restricted to the five taxa under study (notice that some of the trees are not fully resolved—a reflection of the use of strict consensus on multiple trees). When reconciling a gene tree that is not fully resolved with a species phylogeny, we considered *all* possible full resolutions of the gene tree, and considered the resolution that resulted in the best score.

To account for the model parameterization in the likelihood computation, we computed the values of three information criteria, AIC by [11], AICc by [12] and BIC by [13] , in order to account for the number of parameters and allow for model selection.

The AIC measure is defined as:

$$AIC = -2\ln L + 2k, \tag{1}$$

where $\ln L$ is the log likelihood score, and k is the number of parameters. In our case, the number of parameters equals the number of branch lengths being estimated plus the number of hybridization probabilities being estimated.

The AICc measure corrects for finite sample size, and is defined as:

$$AICc = -2\ln L + 2k + \frac{2k(k+1)}{n-k-1}, \tag{2}$$

where $\ln L$ and k are as in the case of AIC, and n is the number of gene trees used to estimated the likelihood score.

Finally, the BIC measure is defined as:

$$BIC = -2\ln L + k\ln n. \tag{3}$$

The lower the values of these criteria, the better the fit of the model to the data.

Table S2. The different topologies inferred by MrBayes and/or PAUP* along with their posterior probabilities (for MrBayes analyses) and frequencies (for PAUP* analyses).

	Topology	Posterior	Frequency
Fully resolved	(Sbay,(Skud,(Smik,(Scer,Spar))));	56.511009	57
	(Skud,(Sbay,(Smik,(Scer,Spar))));	12.874292	2
	(Smik,((Skud,Sbay),(Scer,Spar)));	11.280507	10
	((Skud,Sbay),(Smik,(Scer,Spar)));	9.679648	13
	(Scer,(Spar,(Smik,(Skud,Sbay))));	5.588862	1
	((Smik,(Skud,Sbay)),(Scer,Spar));	5.130724	2
	(Spar,(Scer,(Smik,(Skud,Sbay))));	3.018667	
	(Sbay,((Smik,Skud),(Scer,Spar)));	0.412878	2
	((Sbay,(Smik,(Skud,(Scer,Spar))));	0.314893	1
	(Skud,(Sbay,(Spar,(Scer,Smik))));	0.241837	
	(Skud,((Smik,Sbay),(Scer,Spar)));	0.176712	
	(Skud,(Sbay,(Scer,(Spar,Smik))));	0.142451	
	(Sbay,(Skud,(Spar,(Scer,Smik))));	0.083523	
	(Sbay,(Skud,(Scer,(Spar,Smik))));	0.062064	
	(Smik,(Scer,(Spar,(Skud,Sbay))));	0.062064	1
	((Skud,Sbay),(Spar,(Scer,Smik)));	0.058262	
	(Skud,(Smik,(Sbay,(Scer,Spar))));	0.057062	
	(Scer,(Spar,(Skud,(Smik,Sbay))));	0.053531	
	(Spar,((Skud,Sbay),(Scer,Smik)));	0.033732	
	((Skud,(Smik,Sbay)),(Scer,Spar));	0.033399	
	((Skud,Sbay),(Scer,(Spar,Smik)));	0.0292	
	(Smik,(Spar,(Scer,(Skud,Sbay))));	0.025603	
	(Smik,(Sbay,(Skud,(Scer,Spar))));	0.023735	
	((Sbay,(Smik,Skud)),(Scer,Spar));	0.017867	
	(Smik,(Skud,(Sbay,(Scer,Spar))));	0.016869	
	(Scer,(Smik,(Spar,(Skud,Sbay))));	0.013135	
	(Spar,(Scer,(Skud,(Smik,Sbay))));	0.010198	
	(Spar,(Smik,(Scer,(Skud,Sbay))));	0.009268	
	((Smik,Sbay),(Skud,(Scer,Spar)));	0.008334	
	(Scer,(Spar,(Sbay,(Smik,Skud))));	0.006803	
	(Scer,((Spar,Smik),(Skud,Sbay)));	0.004468	
	((Spar,(Sbay,Skud)),(Scer,Smik));	0.004334	
	((Smik,Skud),(Sbay,(Scer,Spar)));	0.002734	
	(Sbay,(Smik,(Spar,(Scer,Skud))));	0.0026	
	(Spar,(Scer,(Sbay,(Skud,Smik))));	0.002467	
	(Smik,(Sbay,(Spar,(Scer,Skud))));	0.001267	

Table S3. Continuation of Table S2.

	Topology	Posterior	Frequency
Fully resolved	(Skud,(Scer,(Spar,(Smik,Sbay))));	0.001067	
	((Spar,Smik),(Scer,(Skud,Sbay)));	9.36E-4	
	((Smik,Sbay),(Spar,(Scer,Skud)));	6.67E-4	
	(Smik,(Skud,(Spar,(Scer,Sbay))));	4.67E-4	
	(Skud,(Spar,(Scer,(Smik,Sbay))));	4E-4	
	(Skud,(Spar,(Sbay,(Scer,Smik))));	3.33E-4	
	(Sbay,(Scer,(Spar,(Smik,Skud))));	2.0E-4	
	(Smik,(Spar,(Skud,(Scer,Sbay))));	1.34E-4	
	((Smik,Skud),(Spar,(Scer,Sbay)));	1.33E-4	
	(Smik,(Sbay,(Scer,(Spar,Skud))));	6.7E-5	
	((Spar,(Smik,Sbay)),(Scer,Skud));	6.7E-5	
	(Scer,((Spar,Skud),(Smik,Sbay)));	6.7E-5	
	(Skud,((Smik,(Scer,Sbay)),Spar));	6.7E-5	
	(Sbay,(Smik,(Scer,(Spar,Skud))));	6.7E-5	
	((Spar,Sbay),(Skud,(Scer,Smik)));	1.33E-4	
	(Skud,((Spar,Sbay),(Scer,Smik)));	1.33E-4	
	(Sbay,(Spar,(Scer,(Smik,Skud))));	6.7E-5	
	(Spar,((Smik,Skud),(Scer,Sbay)));	6.7E-5	
	(Sbay,(Scer,(Skud,(Spar,Smik))));	6.7E-5	
Partially resolved	((Scer,Spar),Smik,(Skud,Sbay));		4
	(((Scer,Spar),Smik),Skud,Sbay);		3
	((Scer,Spar),Smik,Skud,Sbay);		3
	(((Scer,Spar),Smik,Skud),Sbay);		2
	(Scer,Spar,Smik,(Skud,Sbay));		2
	(((Scer,Spar),Skud,Sbay),Smik);		1
	(Scer,Spar,(Smik,(Skud,Sbay)));		1
	(Scer,Spar,Smik,Skud,Sbay);		1

5 Simulating gene genealogies

We used the ms program [14] to generate synthetic data reflecting six different scenarios that combine hybridization, divergence, and extinction in various ways; these scenarios are depicted by the phylogenetic networks in Fig. S5. In our simulations, all horizontal branches in Fig. S5 had length 0. In all cases, we simulated

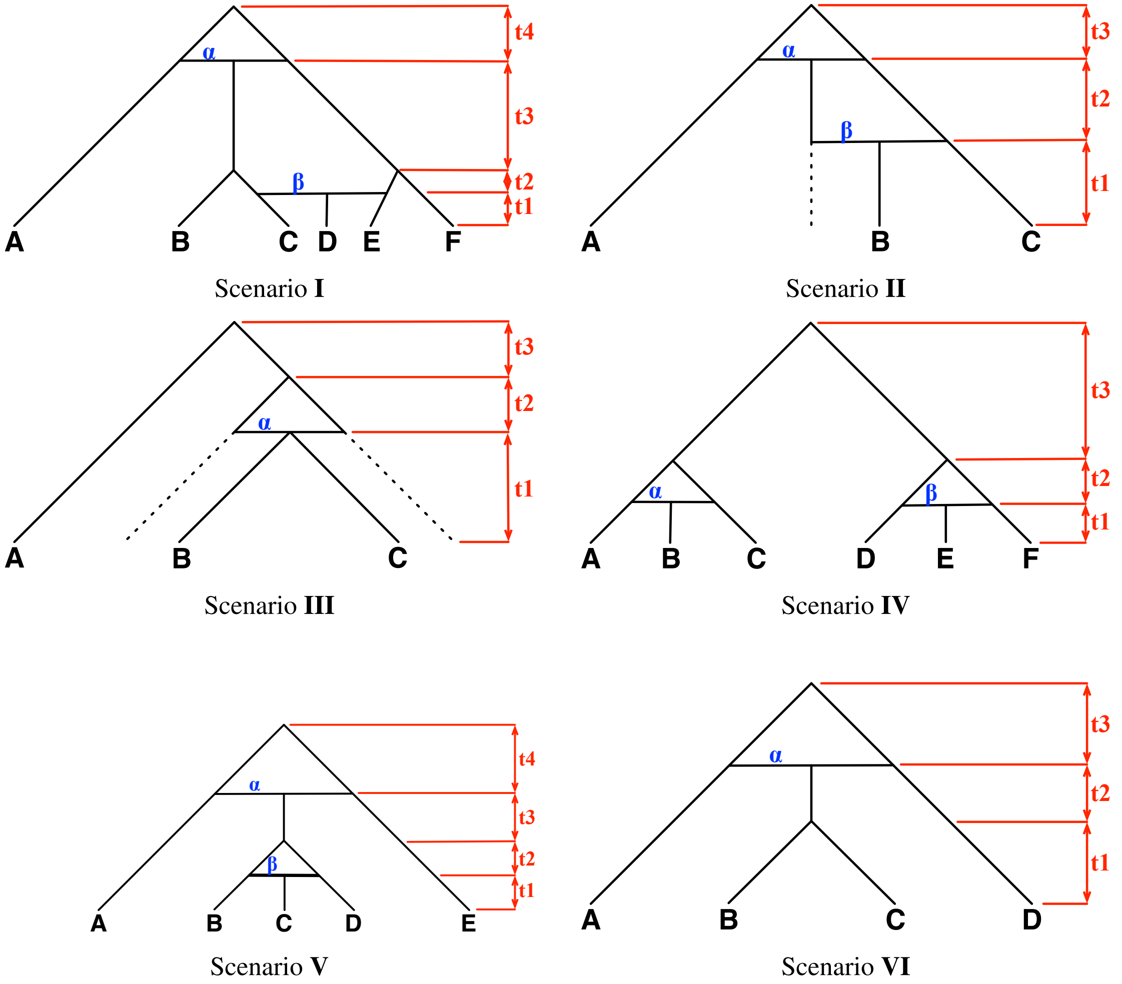

Fig. S5. Phylogenetic networks depicting different hybridization/divergence/extinction scenarios. The α and β parameters denote the proportions (or, probabilities) of alleles that are inherited from the "left" parents of the reticulation nodes ($1 - \alpha$ and $1 - \beta$ denote the proportions of the alleles that are inherited from the "right" parents of the nodes).

$nloci \in \{10, 25, 50, 100, 500, 1000, 2000\}$ loci, for two time intervals: interval 1, which corresponds to $t1 = t2 = t3 = t4 = 1.0$ coalescent units, and interval 2, which corresponds to $t1 = t2 = t3 = t4 = 2.0$ coalescent units. It is important to note that the ms program measures time in $4N_e$ units, where N_e is the effective population size. Since a coalescent unit equals $2N_e$, we used values 0.5 and 1.0 for times in ms to reflect time intervals 1 and 2, respectively. For each setting of parameters, 100 data sets were generated, and averaged results over the 100 data sets were computed.

For scenario **I**, gene genealogies were generated using the command:

ms 6 $nloci$ -T -I 6 1 1 1 1 1 1 -es t1 4 β -ej t1 4 3 -ej t1 7 5 -ej t1+t2 3 2 -ej t1+t2 6 5 -es t1+t2+t3 2 α -ej t1+t2+t3 2 1 -ej t1+t2+t3 8 5 -ej t1+t2+t3+t4

```
5 1
```
for $(\alpha, \beta) \in \{(0.0, 0.5), (0.3, 0.3), (0.5, 0.0), (0.5, 0.5), (0.5, 1.0)\}$.

For scenario **II**, gene genealogies were generated using the command:
```
ms 3 100 -T -I 3 1 1 1 -es t1 2 β -ej t1 4 3 -es t1+t2 2 α -ej t1+t2 2 1 -ej
t1+t2 5 3 -ej t1+t2+t3 3 1
```
for $(\alpha, \beta) \in \{(0.0, 0.5), (0.3, 0.3), (0.5, 0.0), (0.5, 0.5), (0.5, 1.0)\}$.

For scenario **III**, gene genealogies were generated using the command:
```
ms 3 100 -T -I 3 1 1 1 -ej t1 3 2 -es t1+t2 2 α -ej t1+t2+t3 4 2 -ej t1+t2+t3+t4
2 1
```
for $\alpha \in \{0.0, 0.3, 0.5\}$.

For scenario **IV**, gene genealogies were generated using the command:
```
ms 6 100 -T -I 6 1 1 1 1 1 1 -es t1 2 α -ej t1 2 1 -ej t1 7 3 -ej t1+t2 3 1 -es
t1 5 β -ej t1 5 4 -ej t1 8 6 -ej t1+t2 6 4 -ej t1+t2+t3 1 4
```
for $(\alpha, \beta) \in \{(0.0, 0.5), (0.3, 0.3), (0.5, 0.5)\}$.

For scenario **V**, gene genealogies were generated using the command:
```
ms 5 100 -T -I 5 1 1 1 1 1 -es t1 3 β -ej t1 3 2 -ej t1 6 4 -ej t1+t2 4 2 -es
t1+t2+t3 2 α -ej t1+t2+t3 2 1 -ej t1+t2+t3 7 5 -ej t1+t2+t3+t4 5 1
```
for $(\alpha, \beta) \in \{(0.0, 0.5), (0.3, 0.3), (0.5, 0.5)\}$.

For scenario **VI**, gene genealogies were generated using the command:
```
ms 4 100 -T -I 4 1 1 1 1 -ej t1 3 2 -es t1+t2 2 α -ej t1+t2 2 1 -ej t1+t2 5 4
-ej t1+t2+t3 1 4
```
for $\alpha \in \{0.0, 0.3, 0.5\}$.

6 Accuracy of inference

For the four scenarios **I**, **IV**, **V**, and **VI**, the parameters (branch lengths and hybridization probabilities) are identifiable, and we focused on the accuracy of our method for inferring these parameters from samples of gene trees that were simulated as discussed in the previous section. That is, given a sample $\mathcal{G}$ of gene tree topologies, and a phylogenetic network topology W, we solved

$$(\boldsymbol{\lambda}^*, \boldsymbol{\gamma}^*) \leftarrow \mathrm{argmax}_{(\boldsymbol{\lambda},\boldsymbol{\gamma})} P_{W,\boldsymbol{\lambda},\boldsymbol{\gamma}}(\mathcal{G}), \tag{4}$$

where $P_{W,\boldsymbol{\lambda},\boldsymbol{\gamma}}(\mathcal{G})$ is computed based on Equation (2) in the main text.

To infer the hybridization probabilities, we used a grid search of values between 0 and 1 with step length of 0.01. For the branch lengths, we used a grid search of values between 0.1 and 4.0 with step length of 0.1.

The results are shown in Figs. S6—S9 below, and they show very good performance in terms of the accuracy of the parameter values estimated (as compared to the true values used to generate the data).

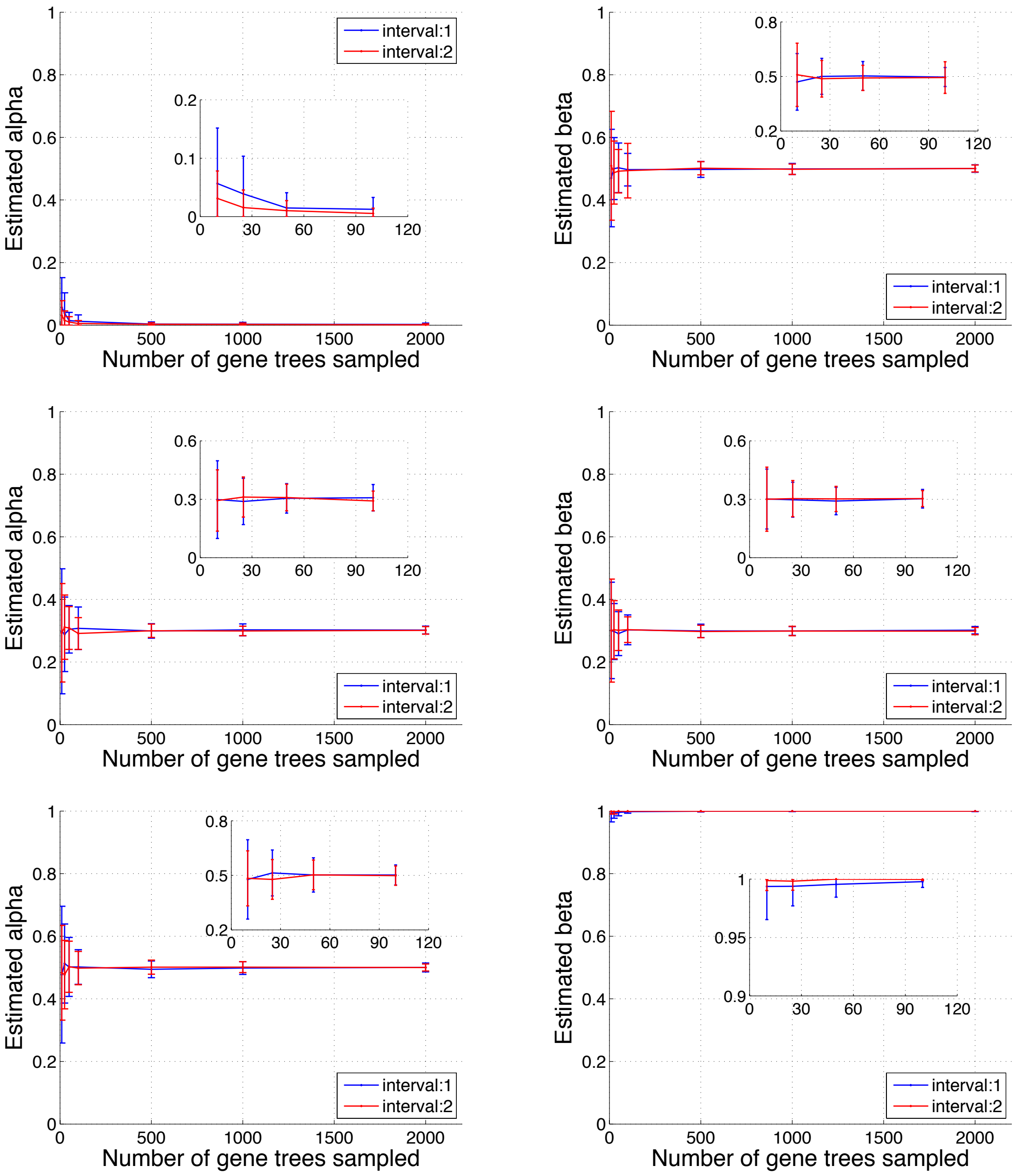

Fig. S6. Estimates of α and β on Scenario **I**. Rows from top to bottom correspond to true (α, β) values of $(0.0, 0.5)$, $(0.3, 0.3)$, and $(0.5, 1.0)$, respectively.

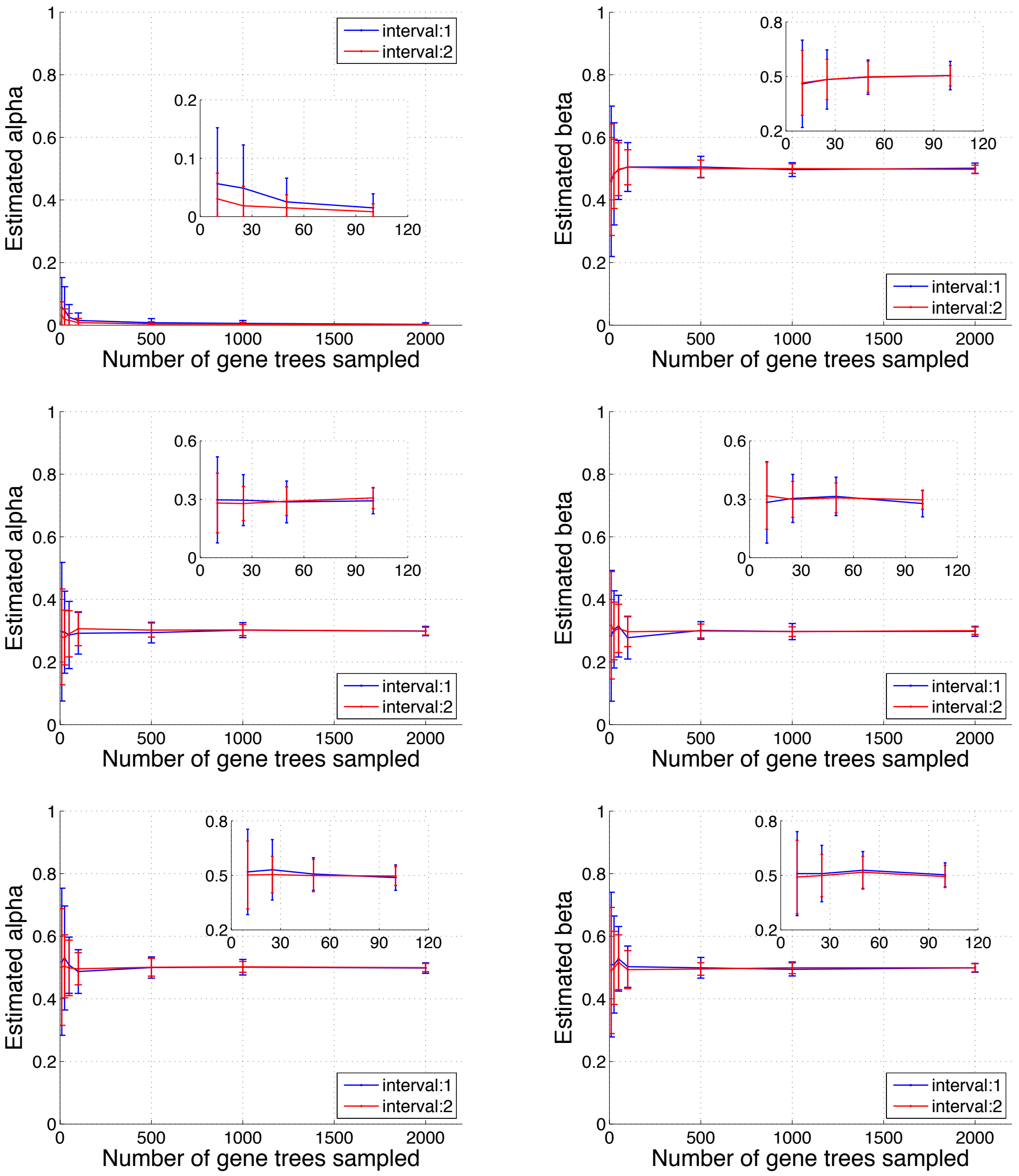

Fig. S7. Estimates of α and β on Scenario **IV**. Rows from top to bottom correspond to true (α, β) values of $(0.0, 0.5)$, $(0.3, 0.3)$, and $(0.5, 0.5)$, respectively.

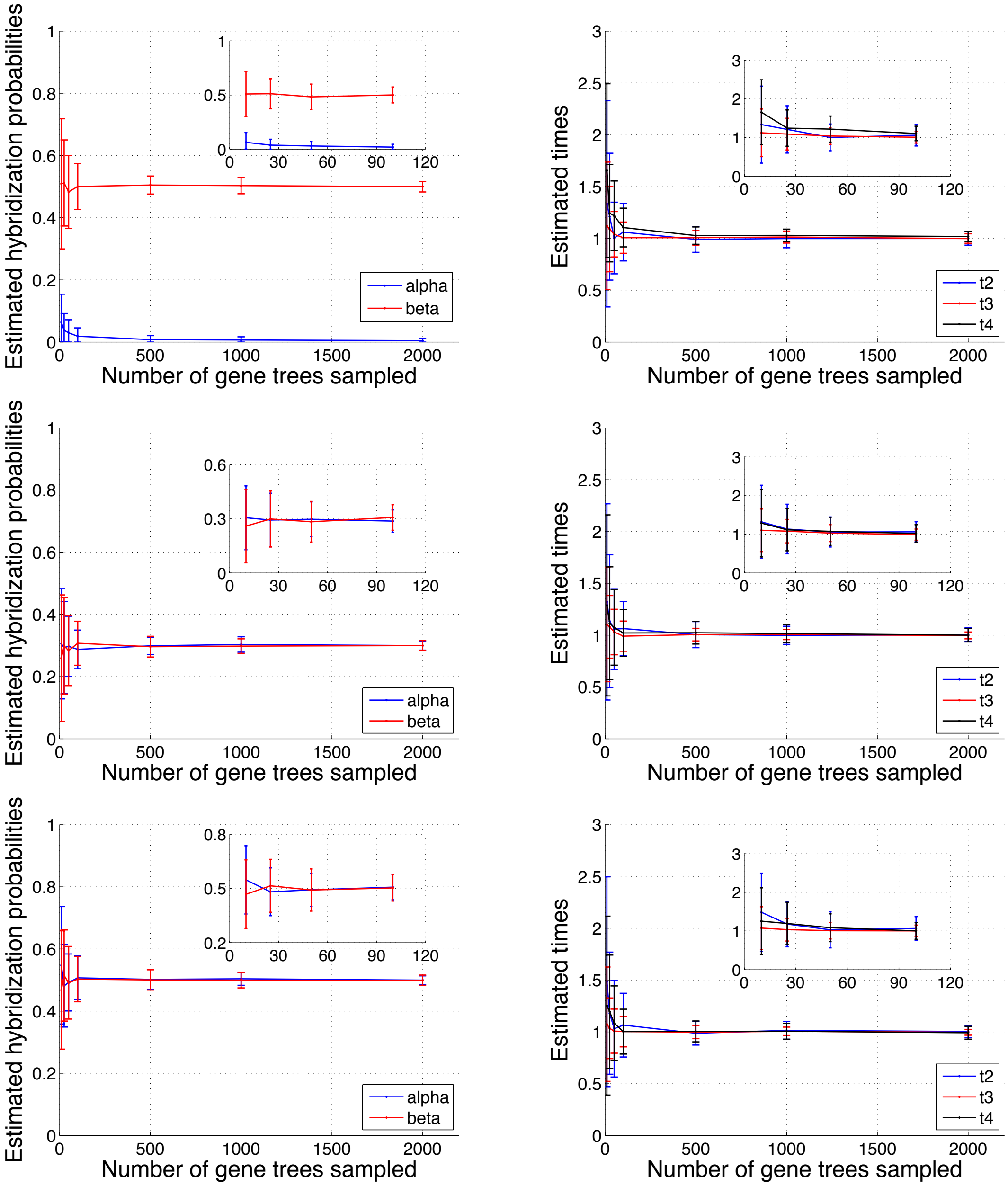

Fig. S8. Estimates of α, β, $t2$, $t3$, and $t4$ on Scenario **V**. Rows from top to bottom correspond to true (α, β) values of $(0.0, 0.5)$, $(0.3, 0.3)$, and $(0.5, 0.5)$, respectively. All plots correspond to true values of $t1 = t2 = t3 = t4 = 1.0$.

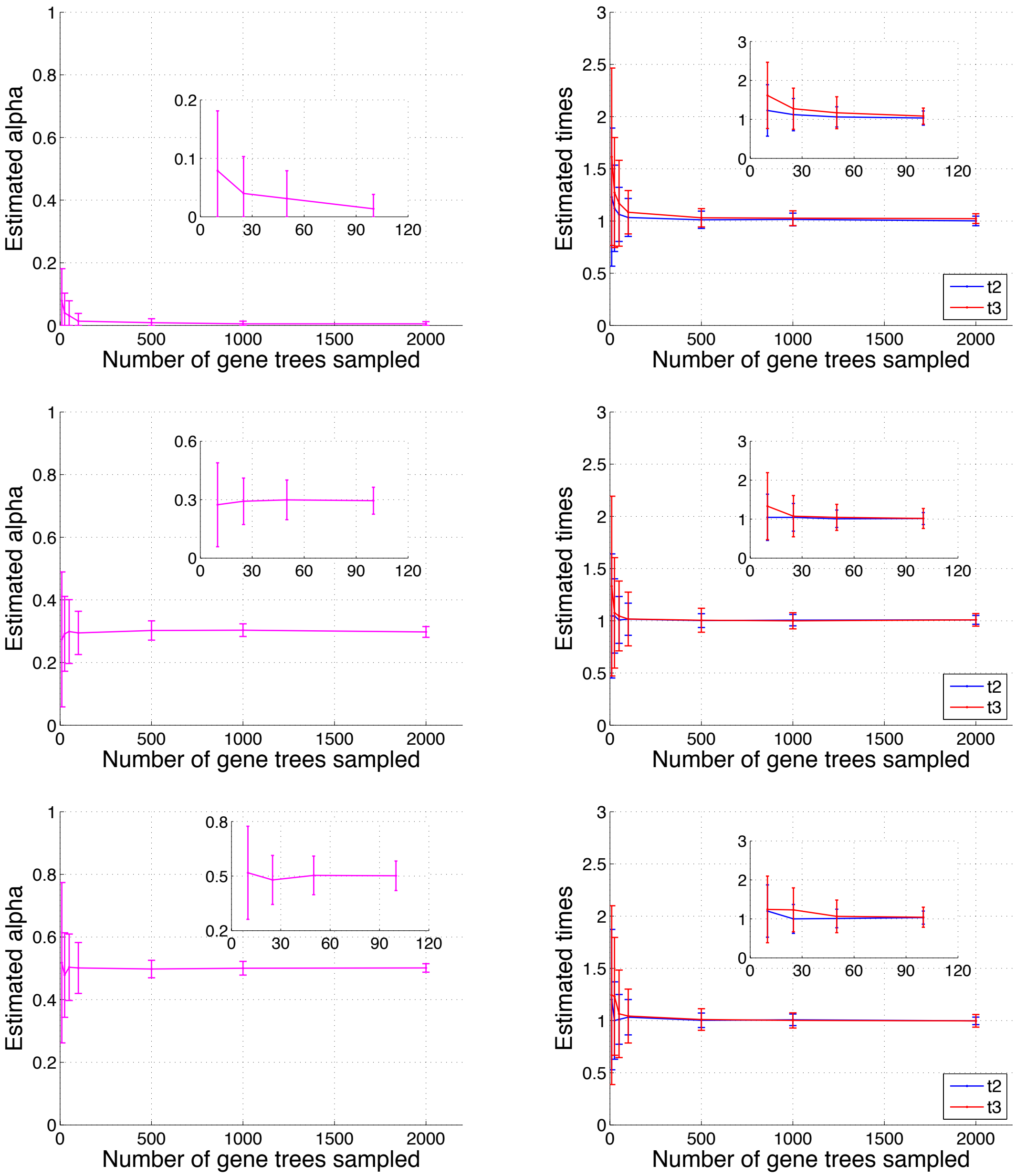

Fig. S9. Estimates of α, $t2$, and $t3$ on Scenario **VI**. Rows from top to bottom correspond to true α values of 0.0, 0.3, and 0.5, respectively. All plots correspond to true values of $t2 = t3 = 1.0$.

7 Identifiability

The results in Fig. S10 show that if we use the correct (true) values of branch lengths, the hybridization probabilities are identifiable, and can be estimated with high accuracy as the number of gene trees sampled increases (the inference procedure is identical to that described in the previous section).

However, if both branch lengths and hybridization probabilities are to be estimated, then issues of unidentifiability arise, as we now show.

Consider the phylogenetic network depicted by Scenario **II** in Fig. S5. Let $\boldsymbol{\lambda}$ be the branch lengths vector with $\lambda_1 \equiv t_1 = s$, $\lambda_2 \equiv t_2 = p$, and $\lambda_3 \equiv t_3 = q$, and let $\boldsymbol{\gamma}$ be the hybridization probabilities vector with $\gamma_1 \equiv \alpha = a$ and $\gamma_2 \equiv \beta = b$. For a given set $\mathcal{G}$ of gene trees, we can obtain other vectors $\boldsymbol{\lambda'}$ and $\boldsymbol{\gamma'}$ such that

$$P_{W,\boldsymbol{\lambda},\boldsymbol{\gamma}}(\mathcal{G}) = P_{W,\boldsymbol{\lambda'},\boldsymbol{\gamma'}}(\mathcal{G}),$$

by setting the branch lengths arbitrarily to $t_1 = s'$, $t_2 = p'$, $t_3 = q'$, and then setting the hybridization probabilities as follows

$$\alpha = -\frac{(e^{p'}-1)(e^{q}-1)abe^{p+q'}}{(e^{q'}-1)(e^{p+q} - be^{p+p'+q'} - e^{p'+q'} + be^{p'+q'})}$$

and

$$\beta = -\frac{(e^{p+q} - be^{p+p'+q'} - e^{p'+q'} + be^{p'+q'})e^{-(p+q)}}{e^{p'}-1}.$$

For example, if we use $p = 2.0$, $q = 2.0$, $a = 0.5$, $b = 0.5$, $p' = 1.7$, $q' = 1.7$, and then set $\alpha = 0.9088149157446168$ and $\beta = 0.29101947060819205$ (based on the above two formulas), then we obtain the same probability of any set of gene trees on the phylogenetic network of Scenario **II** in Fig. S5.

If we sample two alleles per species B (and a single or more alleles per each of the two species A and C), this lack of identifiability case disappears, since now the number of gene tree topologies is greater than the number of parameters being estimated. However, in practice, the value of t_1 does affect the identifiability of the parameter values, since the larger it is, the higher the probability that the two alleles sampled from B would coalesce and give a signal similar to that provided by a single allele. This point is illustrated by the results shown in Fig. S11.

To produce these results, we parameterized the phylogenetic network of Scenario **II** above with two different sets of values:

- network1: $t_2 = t_3 = 2.0$, $\alpha = \beta = 0.5$.
- network2: $t_2 = t_3 = 1.7$, $\alpha = 0.9088149157446168$ and $\beta = 0.29101947060819205$.

As discussed above, the probability of each of the three gene tree topologies ((A,B),C), ((A,C),B), and ((B,C),A), is the same under both networks. However, we now consider the case where two alleles from B are sampled. In this case, there are 15 different gene tree topologies, which can be grouped into 9 categories, where all gene tree topologies within the same category have identical probabilities, regardless of the species phylogeny:

1. (B2,((B1,C),A)) and (B1,((B2,C),A))
2. (B1,(C,(B2,A))) and (B2,(C,(B1,A)))
3. (C,(B1,(B2,A))) and (C,(B2,(B1,A)))
4. ((B1,C),(B2,A)) and ((B2,C),(B1,A))
5. (A,(B2,(B1,C))) and (A,(B1,(B2,C)))
6. (A,(C,(B1,B2)))
7. (B1,(B2,(A,C))) and (B2,(B1,(A,C)))

8. (C,(A,(B1,B2)))

9. ((B1,B2),(A,C))

The probabilities of each of these 9 gene tree topologies (we choose one gene tree topology per category), as a function of the value of t_1 are shown in Fig. S11.

Clearly, the two networks exhibit the gene tree topologies with different probabilities, when $t_1 = 0.25$. However, the gap between the probabilities starts closing as the value of t_1 increases. When $t_1 = 4.0$ or 8.0, the gaps are too small to be even observed in any realistic data set (of a few thousand loci). At these branch lengths, the three topologies with non-negligible probabilities are the ones of categories 6, 8, and 9, which have the two alleles of B coalesce before either of them coalesce with alleles of the other two species.

In other words, while sampling two alleles from B help ameliorate the identifiability issue, a relatively large sample (in terms of the number of loci) needs to be used, and the the time between hybridization and the subsequent divergence must not be too large, for methods to uniquely identify the parameter values.

Furthermore, in the special case where $\alpha = 0.0$, a phylogenetic tree, with appropriate branch lengths can be found, to fit the data exactly with the same probability that the phylogenetic network would. Consider the phylogenetic network N in Fig. S12(left), which reflects Scenario **II** in Fig. S5 in the case where $\alpha = 0.0$.

Let $\boldsymbol{\lambda}$ be the branch lengths vector with $\lambda_1 \equiv t_1$, $\lambda_2 \equiv t_2$, and $\lambda_3 \equiv t_3$, and let $\boldsymbol{\gamma}$ be the hybridization probabilities vector with $\gamma_1 \equiv \beta$. Now, consider the phylogenetic tree T in Fig. S12(right). Then, if we set t as a function of β, t_2, and t_3, as follows:

$$t(\beta, t_2, t_3) = -\ln(\beta e^{t_2} + 1 - \beta) + t_2 + t_3,$$

then,

$$P_{N,\boldsymbol{\lambda},\boldsymbol{\gamma}}(\mathcal{G}) = P_{T,t}(\mathcal{G})$$

for any set $\mathcal{G}$ of gene trees.

This result shows (as illustrated in Fig. 2 in the main text) that as t_2 increases, the value of t becomes unaffected by t_2, and that increasing t proportionally to the increase in t_3 always maintains identical probabilities of gene trees under both phylogenies in Fig. S12, as reflected by the derivatives:

$$\frac{\partial t}{\partial t_2} = 1 - \frac{\beta e^{t_2}}{\beta e^{t_2} + 1 - \beta} = 1 - \frac{1}{1 + \frac{1-\beta}{\beta e^{t_2}}}$$

and

$$\frac{\partial t}{\partial t_3} = 1.$$

Clearly,

$$\lim_{t_2 \to \infty} \frac{\partial t}{\partial t_2} = 0.$$

Let us now consider the phylogenetic network of Scenario **III** in Fig. S5. In this case, both species involved in the hybridization are extinct. Surprisingly, the results in Fig. S13 show that if we use the correct (true) values of branch lengths, the hybridization probability α is identifiable, and can be estimated with high accuracy as the number of gene trees sampled increases.

However, if both branch lengths and hybridization probability are to be estimated, then issues of non-identifiability arise, as we now show. Let $\boldsymbol{\lambda}$ be the branch lengths vector with $\lambda_1 \equiv t_1 = s$, $\lambda_2 \equiv t_2 = p$, and $\lambda_3 \equiv t_3 = q$, and let $\boldsymbol{\gamma}$ be the hybridization probabilities vector with $\gamma_1 \equiv \alpha = a$. For a given set $\mathcal{G}$ of gene trees, we can obtain other vectors $\boldsymbol{\lambda'}$ and $\boldsymbol{\gamma'}$ such that

$$P_{W,\boldsymbol{\lambda},\boldsymbol{\gamma}}(\mathcal{G}) = P_{W,\boldsymbol{\lambda'},\boldsymbol{\gamma'}}(\mathcal{G}),$$

by setting the hybridization probability arbitrarily to $\alpha = a'$ and the branch lengths arbitrarily to $t_1 = s'$, $t_3 = q'$, and

$$t_2 = -\ln \frac{2a'e^{p+q}(a'-1) + 2ae^{p+q'}(1-a) + 2ae^{q'}(a-1) + e^{q'}}{e^{q'}(2a'^2 + 1 - 2a')} + p + q - q'.$$

For example, if we use $p = 1.0$, $q = 2.0$, $a = 0.8$, $a' = 0.1$, $q' = 1.8$, and then set $p' = 1.050498643$ (based on the above formula), then we obtain the same probability of any set of gene trees on the phylogenetic network of Scenario **III** in Fig. S5.

Furthermore, a phylogenetic tree, with appropriate branch lengths can be found, to fit the data exactly with the same probability that the phylogenetic network would. Let $\boldsymbol{\lambda}$ be the branch lengths vector with $\lambda_1 \equiv t_1$, $\lambda_2 \equiv t_2$, and $\lambda_3 \equiv t_3$, and let $\boldsymbol{\gamma}$ be the hybridization probabilities vector with $\gamma_1 \equiv \alpha$. Now, consider the phylogenetic tree T in Fig. S12(right). Then, if we set t as a function of α, t_2, and t_3, as follows:

$$t(\alpha, t_2, t_3) = -\ln(2\alpha^2 + 2\alpha e^{t_2} - 2\alpha^2 e^{t_2} + 1 - 2\alpha) + t_2 + t_3 \tag{5}$$

then,

$$P_{N,\boldsymbol{\lambda},\boldsymbol{\gamma}}(\mathcal{G}) = P_{T,t}(\mathcal{G})$$

for any set $\mathcal{G}$ of gene trees. See Fig. S14 for values of $t(\alpha, t_2, t_3)$.

This result shows that as t_2 increases, the value of t becomes unaffected by t_2, and that increasing t proportionally to the increase in t_3 always maintains identical probabilities of gene trees under both the phylogenetic network of Scenario **III** and the phylogenetic tree in Fig. S12, as reflected by the derivatives:

$$\frac{\partial t}{\partial t_2} = 1 - \frac{1}{1 + \frac{1}{e^{t_2}}\left(\frac{1}{2\alpha(1-\alpha)} - 1\right)}$$

and

$$\frac{\partial t}{\partial t_3} = 1.$$

Clearly,

$$\lim_{t_2 \to \infty} \frac{\partial t}{\partial t_2} = 0.$$

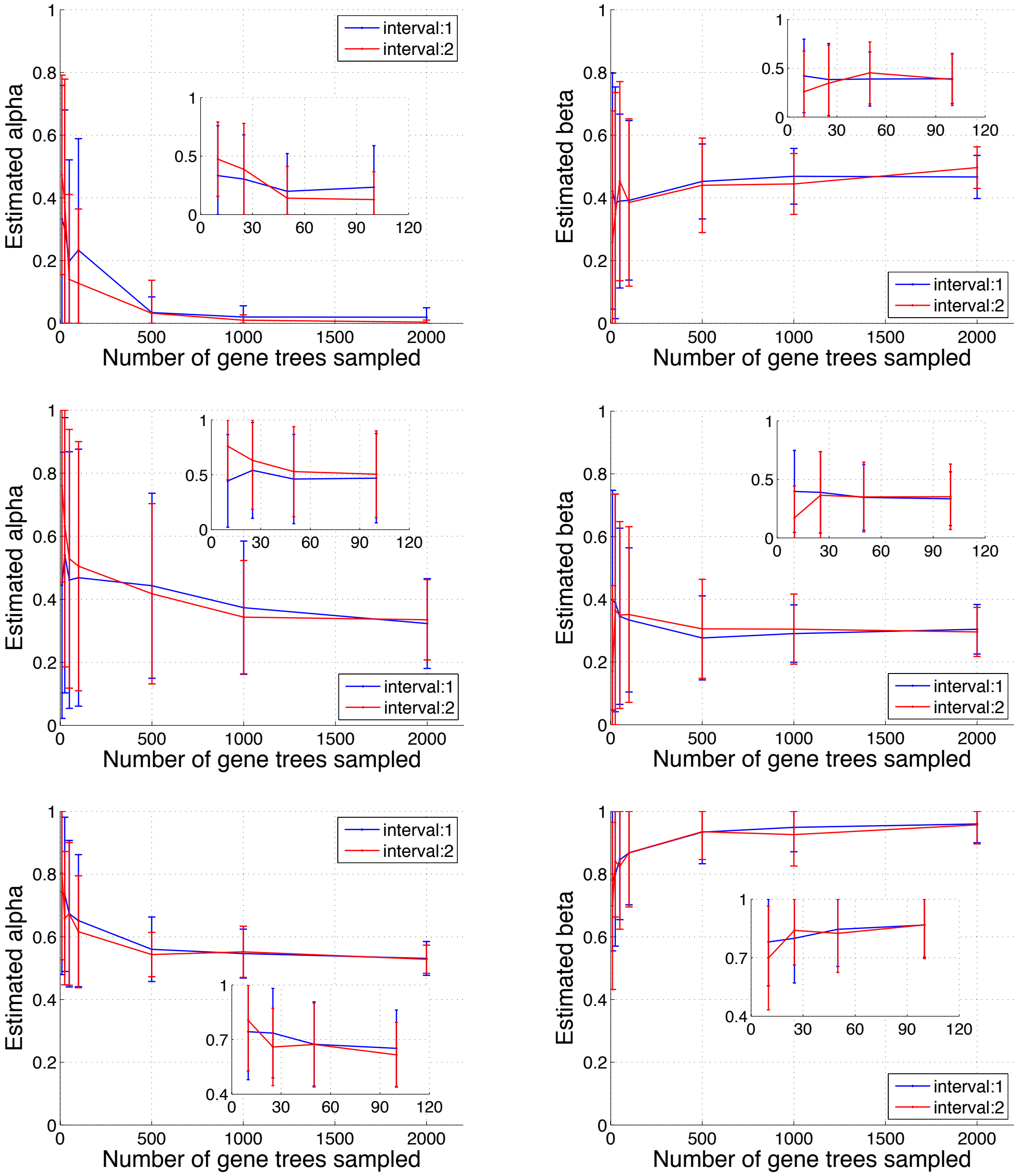

Fig. S10. Estimates of α and β on Scenario **II**. Rows from top to bottom correspond to true (α, β) values of $(0.0, 0.5)$, $(0.3, 0.3)$, and $(0.5, 1.0)$, respectively.

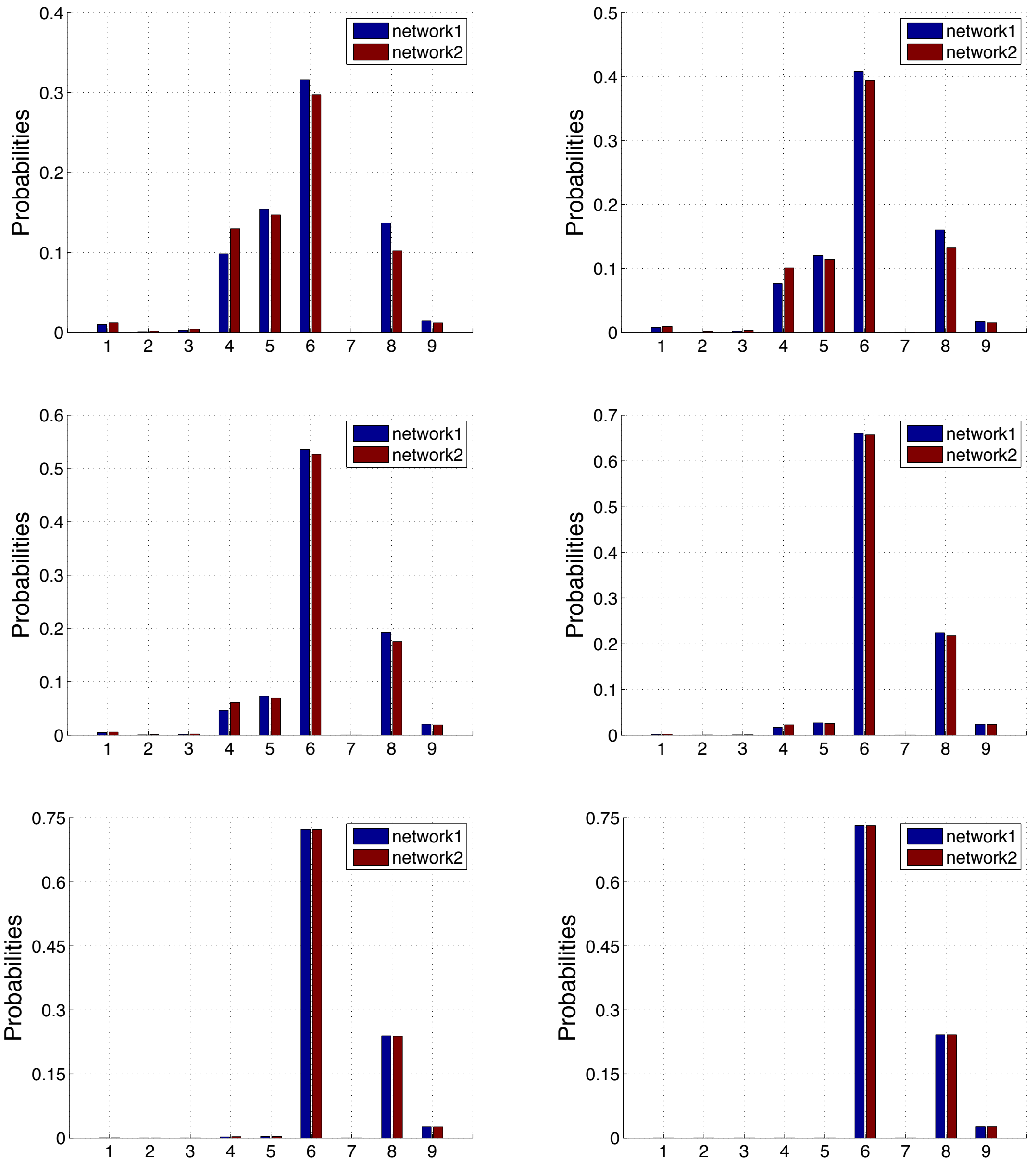

Fig. S11. The probabilities of the 9 different gene tree topologies (when a single allele is sampled from each of two species A and C, and two alleles are sampled from species B) on the two phylogenetic networks obtained by parameterizing the values of α, β, t_2 and t_3 differently for Scenario **II**; see text. Left to right, top to bottom: $t_1 = 0.25, 0.5, 1.0, 2.0, 4.0,$ and 8.0, respectively.

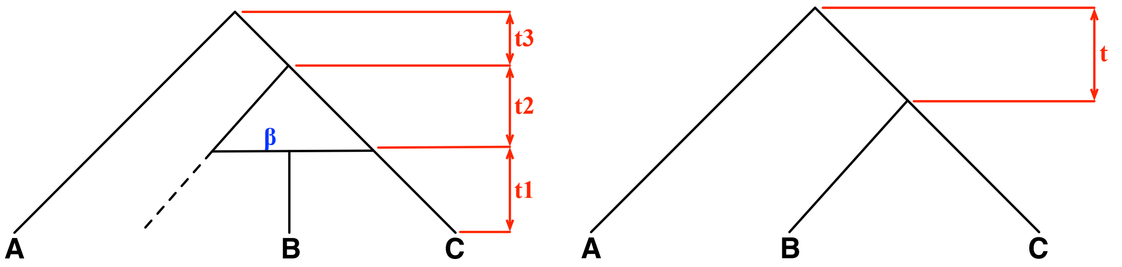

Fig. S12. (Left) A phylogenetic network with one of the parents of the hybrids being extinct. (Right) A phylogenetic tree with divergence time t between the two speciation events.

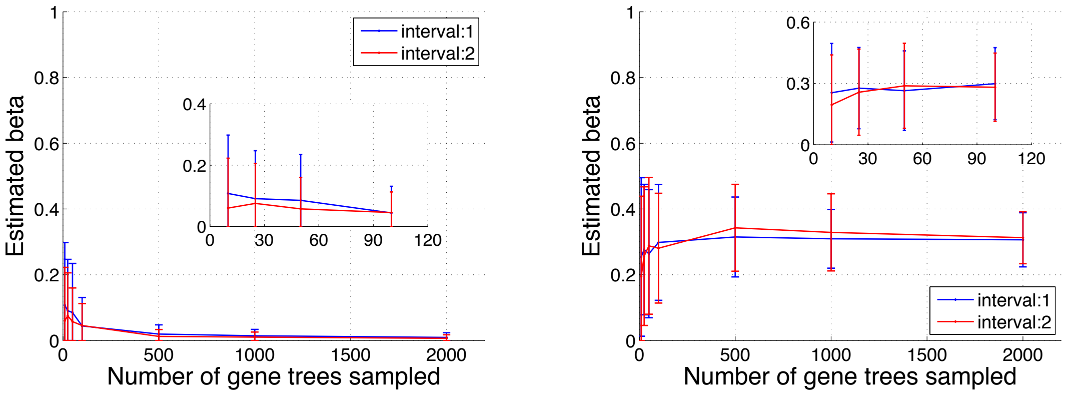

Fig. S13. Estimates of α on Scenario **III**. (Left) $\alpha = 0.0$; (right) $\alpha = 0.3$.

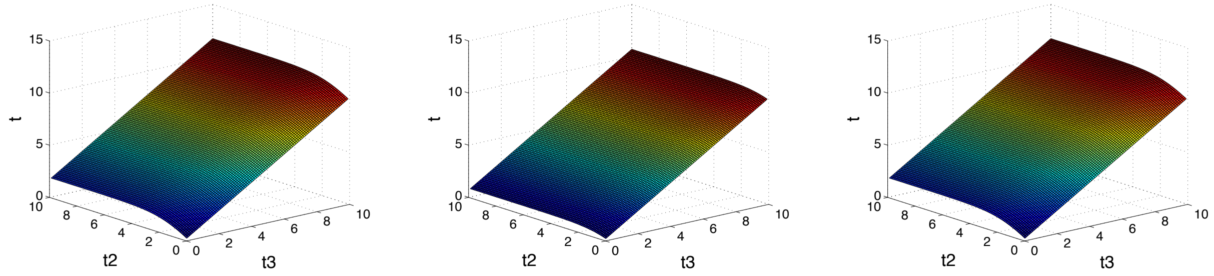

Fig. S14. Values of $t(\alpha, t_2, t_3)$ based on Equation (5); from left to right: $\alpha = 0.1$, 0.5, and 0.9, respectively.

8 References

1. Huson D, Rupp R, Scornavacca C (2010) Phylogenetic Networks: Concepts, Algorithms and Applications. New York: Cambridge University Press.

2. Nakhleh L (2010) Evolutionary phylogenetic networks: models and issues. In: Heath L, Ramakrishnan N, editors, The Problem Solving Handbook for Computational Biology and Bioinformatics, New York: Springer. pp. 125-158.

3. Huber K, Oxelman B, Lott M, Moulton V (2006) Reconstructing the evolutionary history of polyploids from multilabeled trees. Molecular Biology and Evolution 23: 1784-1791.

4. Degnan J, Salter L (2005) Gene tree distributions under the coalescent process. Evolution 59: 24-37.

5. Rosenberg N (2007) Counting coalescent histories. Journal of Computational Biology 14: 360-377.

6. Than C, Ruths D, Innan H, Nakhleh L (2007) Confounding factors in HGT detection: Statistical error, coalescent effects, and multiple solutions. J Comput Biol 14: 517-535.

7. Rokas A, Williams B, King N, Carroll S (2003) Genome-scale approaches to resolving incongruence in molecular phylogenies. Nature 425: 798–804.

8. Than C, Nakhleh L (2009) Species tree inference by minimizing deep coalescences. PLoS Computational Biology 5: e1000501.

9. Huelsenbeck JP, Ronquist F (2001) MRBAYES: Bayesian inference of phylogenetic trees. Bioinformatics 17: 754-755.

   KEY: HuelsenbeckAndRonquist01
   ANNOTATION:

10. Swofford DL (1996). PAUP*: Phylogenetic analysis using parsimony (and other methods). Sinauer Associates, Underland, Massachusetts, Version 4.0.

11. Akaike H (1974) A new look at the statistical model identification. IEEE Trans Automat Contr 19: 716-723.

12. Burnham K, Anderson D (2002) Model selection and multi-model inference: a practical-theoretic approach. New York: Springer Verlag, 2nd edition.

    KEY: BurnhamAnderson02
    ANNOTATION:

13. Schwarz G (1978) Estimating the dimension of a model. Annals of Statistics 6: 461-464.

14. Hudson RR (2002) Generating samples under a Wright-Fisher neutral model of genetic variation. Bioinformatics 18: 337-338.

    KEY: Hudson02
    ANNOTATION:
